# Emerging nanotechnology approaches for sustainable water treatment and heavy metals removal: a comprehensive review

**DOI:** 10.1039/d5ra06914a

**Published:** 2025-10-28

**Authors:** Erfan Burhan Hussein, Farouk Abdullah Rasheed, Ahmed Salih Mohammed, Kawan F. Kayani

**Affiliations:** a Department of Water Resources Engineering, College of Engineering, University of Sulaimani Sulaymaniyah 46001 Kurdistan Region Iraq erfan.hussein@univsul.edu.iq farouk.rasheed@univsul.edu.iq; b Department of Civil Engineering, College of Engineering, American University of Iraq, Sulaimani (AUIS) Sulaymaniyah 46001 Kurdistan Region Iraq ahmed.salih@auis.edu.krd; c Department of Chemistry, College of Science, University of Sulaimani Sulaymaniyah 46001 Kurdistan Region Iraq kawan.nasralddin@univsul.edu.iq

## Abstract

Water pollution is a significant worldwide concern, caused mainly by industrialization, urban development, and inadequate waste management, leading to the release of harmful pollutants, especially heavy metals, into water bodies. These pollutants provide significant risks to ecological and public health owing to their toxicity, persistence, and bioaccumulative properties. Traditional water and wastewater treatment methods often inadequately remove these persistent pollutants due to challenges in selectivity, efficiency, and flexibility. Nanotechnology has grown as an effective and innovative method for eliminating pollutants, providing improved materials with distinctive physicochemical characteristics, including large surface area, increased reactivity, and functional tunability. The study systematically examines the utilization of nanotechnology in water and wastewater treatment, focusing on the adsorption-based elimination of heavy metal ions. The article begins with a summary of conventional treatment techniques and their limitations, then addresses adsorption principles, including physisorption and chemisorption processes. The review classifies and critically evaluates various types of nanoadsorbents, such as carbon-based nanocomposites, zeolites, metal and metal oxide nanoparticles, silica nanomaterials, polymer-based materials, metal–organic frameworks, and layered double hydroxides, emphasizing their efficacy under different operational conditions. Furthermore, essential nanomaterial characterization methods are introduced to assess structure–function relationships. This study provides an essential overview for academics and practitioners seeking to develop efficient, sustainable, and cost-effective nanomaterials for advanced water treatment systems by including current advancements and comparative data.

## Abstract 2

Comprehensive review of adsorption mechanisms and nanoadsorbents enabling sustainable water purification and heavy metal remediation.

## Graphical abstract

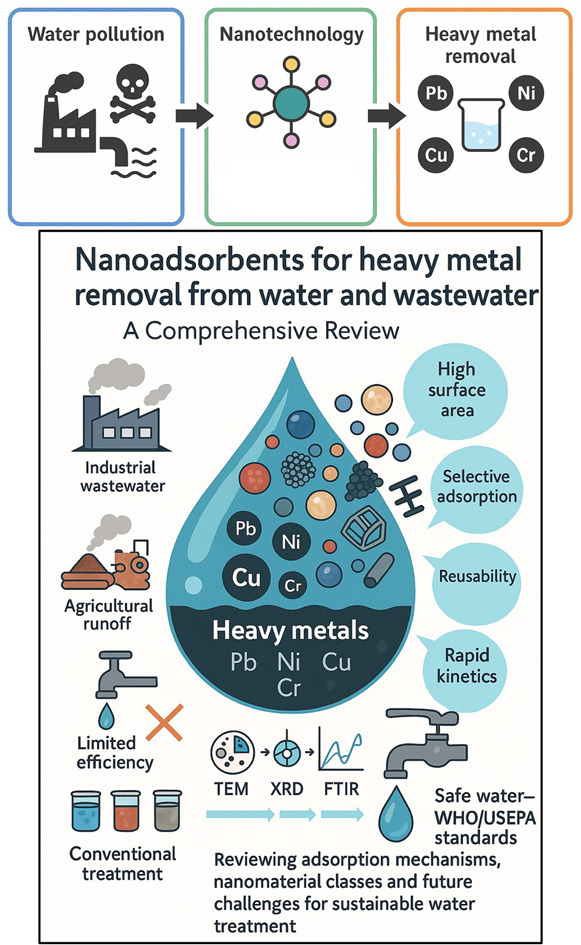

## 1. Introduction

**Fig. 1:**
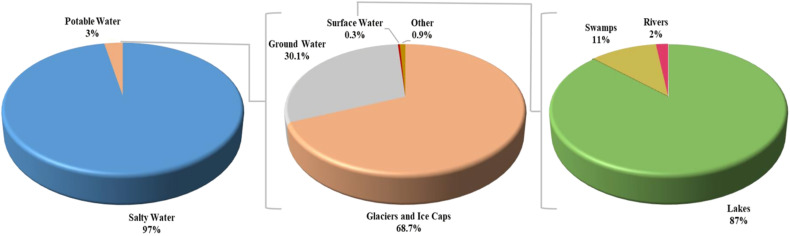
Global distribution of water on the Earth's surface.^19,20^

Water is the fundamental component necessary for every living thing on the planet and a valuable resource for the development of human society.^1^ Ensuring the consistent and dependable availability of uncontaminated and accessible water is widely recognized as an essential purpose of humanistic efforts and continues to be a significant worldwide obstacle. Even though water covers over 71 percent of the Earth's surface, the amount of clean water accessible for utilization remains extremely limited, and the need for water is rising due to the expansion of industrial activity and growing populations.^2^Fig. 1 depicts the global distribution of water. The primary concern associated with rising water consumption is providing potable water to the necessary fields. In addition to its domestic applications for consumption and use in cooking, pure water is also a crucial resource for various emerging businesses, including food, pharmaceuticals, electronics, and medical areas. However, the accessible freshwater reservoir faces complexity due to diminishing quantities caused by global warming, population growth, and stricter health regulations. Research by the World Health Organization (WHO) reveals that 2.1 billion people lack access to potable water sources, and over 700 million people do not have access to the essential daily demand for potable water. Moreover, approximately 4 billion individuals globally experience water shortages for at least one month yearly.^2–5^ Overall, eighty percent of health issues globally can be traced back to a contaminated water supply, directly or indirectly. Additionally, nineteen percent of all human deaths can be attributed to diseases associated with pathogenic microbes present in water.^6^ Furthermore, an alarming number of two million individuals die of diarrheal diseases annually as a direct consequence of the absence of any enhanced sanitary system, and each day, approximately 5000–6000 children lose their lives as a result of diarrhea, a water-based issue. Therefore, an uninterrupted provision of purified water is crucial for our everyday consumption. According to WHO guidelines, drinking water should have undetectable levels of fecal and total coliforms in any 100 mL water sample. The paper, plastic, and metal sectors also rely on providing uncontaminated water for their manufacturing processes. Therefore, individuals can reduce water consumption by gaining a deeper awareness of how water is used and recycled in the production of goods.^4,7–9^ Polluted water not only has adverse effects on the lives of people but also has harmful effects on biodiversity and ecosystems.^10^ It is crucial to implement new regulations for water usage in response to the growing strain on water resources, the unpredictable availability of clean water, and the impact of global climate change. The many demands placed on water systems necessitate the utilization or reutilization of alternate water sources, such as saltwater, stormwater, brine water, and recycled household water, particularly in regions with a history of water scarcity.^11,12^

In addition to these global statistics, several practical cases further illustrate the severity of water pollution. For instance, the Flint water crisis in the United States highlighted the devastating health consequences of lead-contaminated drinking water on local communities.^13^ Chronic exposure to heavy metals such as arsenic, lead, and cadmium through contaminated water has been strongly associated with gastrointestinal disorders and increased cancer risks, particularly rectal and colorectal cancers.^14^ In Bangladesh, high levels of naturally occurring arsenic in groundwater continue to affect millions of people, with epidemiological data linking this exposure to gastrointestinal cancers and other severe health effects.^14–17^ Similarly, in China, long-term exposure to contaminated water containing nitrates, polycyclic aromatic hydrocarbons, and heavy metals has been associated with elevated incidences of digestive system cancers.^14,18^ These practical examples underscore the urgent need for effective and sustainable treatment strategies to mitigate the health and environmental impacts of water pollution.

As a result of the environmental concerns caused by water contamination, many scientists have focused their attention on developing innovative methods for treating wastewater,^21^ and governments, non-governmental organizations, and environmentalists have established strict regulations and standards to promote environmental conservation and the principles of reuse, reduce, and recycle.^22^ Various methods have been created so far to reduce the release of wastewater and lessen the risks associated with contaminants. These methods include coagulation/flocculation, membrane filtration, adsorption, oxidation, chemical precipitation, and biological treatment.^23,24^ Wastewater treatment often incurs substantial expenses due to the necessity of efficiently eliminating the contaminants in the wastewater to render the water safe and appropriate for reuse. Nevertheless, the planning and development of these wastewater treatment methods primarily focus on investigating effluents and treatment standards, disregarding their potential effects on the entire treatment process and sustainability.^25^ Current treatments do not eliminate contaminants; instead, they either concentrate them or transform them into other forms.^26^

**Fig. 2:**
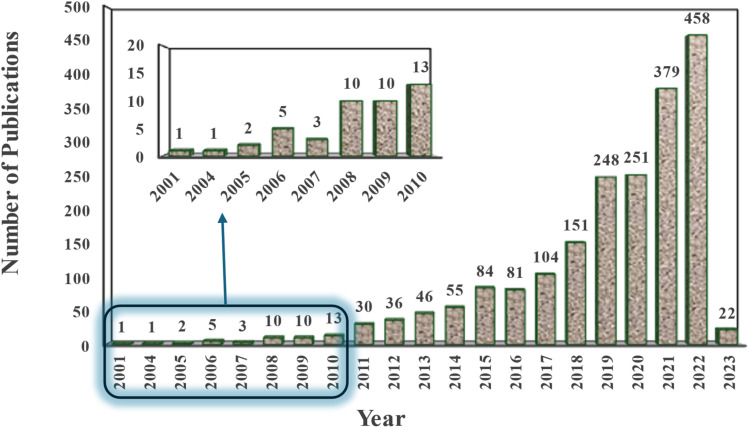
Quantity of published research on nanomaterials in wastewater treatment (Scopus Database).^38^

**Fig. 3:**
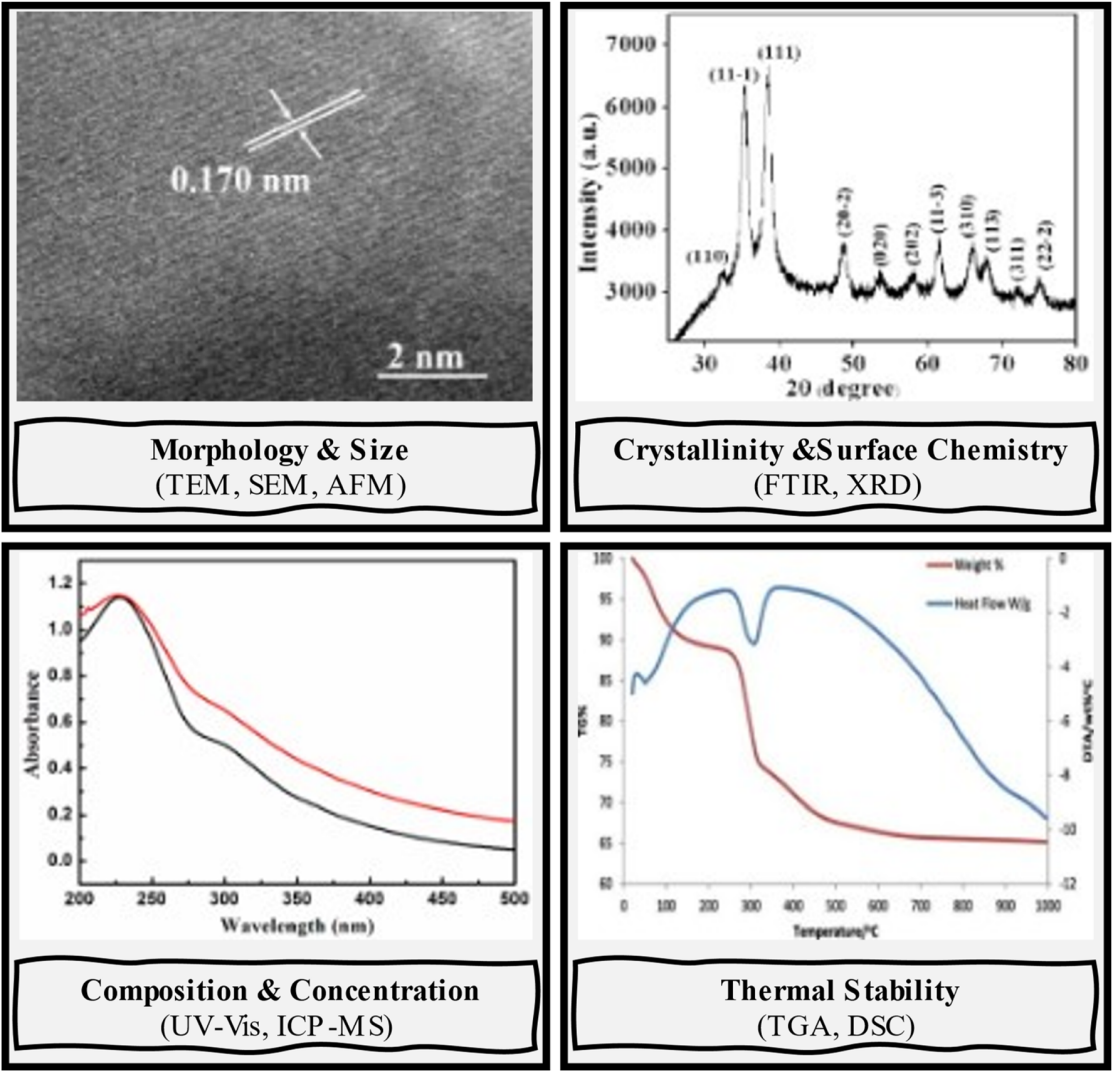
Representative characterization techniques used for nanomaterials.

The progress in nanotechnology presents several possibilities for developing cost-effective and eco-friendly water supply methods. Nanomaterials are anticipated to possess diverse characteristics that provide cost-efficient and highly efficient methods for treating wastewater and water. These techniques would depend less on complex infrastructure for wastewater treatment and could be used with other water treatment processes such as adsorption, coagulation, membrane technology, and photocatalysis.^11,27^ Nanotechnology-based approaches for water and wastewater treatment have emerged as promising solutions to overcome key challenges in conventional treatment techniques. These advanced technologies provide innovative and efficient mechanisms for pollutant removal, enabling the cost-effective utilization of alternative water sources to enhance water availability. The increasing quantity of scientific articles in this field demonstrates the growing interest and recognition of nanotechnology's potential in addressing complex water pollution issues (as shown in Fig. 2). Alongside advances in synthesis, comprehensive characterization of nanomaterials is essential to validate their unique physiochemical properties and ensure their suitability for water and wastewater treatment applications. Different techniques provide complementary insights: microscopy-based methods (*e.g.*, SEM, TEM, AFM) reveal particle size, morphology, and crystal structure; spectroscopy methods (*e.g.*, FTIR, UV-Vis, XPS) identify surface functional groups and electronic state; diffraction and scattering methods (*e.g.*, XRD, SAXS) confirm crystallinity and phase composition; while surface and stability analyses (*e.g.*, BET, zeta potential, DLS) quantify porosity, surface area, dispersion behavior, and colloidal stability.^28–31^Fig. 3 illustrates the main characterization techniques commonly employed in nanomaterial research and the properties they reveal. Including these techniques at the early stage of material evaluation is crucial, as particle size, morphology, surface chemistry, and stability directly govern adsorption performance, regeneration potential, and environmental safety.

This article provides a comprehensive review of recent advancements in nanotechnologies applications for water and wastewater treatment. It highlights the potential of nanomaterials to overcome the limitations of conventional treatment methods by offering enhanced efficiency, selectivity, and reusability. A detailed comparison between traditional techniques and nanotechnology-based approaches is presented to emphasize the superiority of nanoscale materials in pollutant removal. The review further examines the synthesis, structural properties, and performance of various nanoparticles and nanocomposites, particular focusing on their mechanisms of decontamination. Special attention is given to nanoadsorbents for the removal of heavy metals, with discussions on adsorption processes and the influence of key operational parameters such as solution pH, temperature, contact time, initial metal ion concentration, and adsorbent dosage. Overall, this study underscores the significance of nanotechnology as a sustainable and efficient alternative for water purification. Moreover, recent evidence demonstrating the continued rise in industrial effluent discharge and associated heavy metal pollution highlights the urgent need to develop advanced and cost-effective treatment strategies.^32–34^ Numerous research groups have investigated diverse methods for treating heavy metal-contaminated wastewater using a wide range of materials.^35–37^ This review consolidates the most recent progress in employing nanomaterials for heavy metal removal, emphasizing their superior adsorption capacity, regeneration potential, and environmental compatibility compared to conventional adsorbents. The article elaborates on the fundamental principles of nanotechnology, interaction mechanisms between nanomaterials and heavy metal ions, and the types and performance of different nanomaterials utilized for remediation. Furthermore, it identifies knowledge gaps and future perspectives for optimizing nanomaterial design, improving recyclability, and ensuring environmental safety. To the best of our knowledge, this review provides one of the most comprehensive and up-to-date analyses of nanomaterial-based approaches for heavy metal removal, serving as a valuable reference for both researchers and practitioners in developing sustainable water treatment technologies.

## 2. Strategies applied for wastewater treatment and water decontamination

Water contamination harms the ecosystem and can also be a source of air pollution, severely impacting human health: water pollution damages both the economic development and social standing of affected cultures or nations.^39^ Water becomes contaminated when undesirable substances seep into water resources, making them unsafe for drinking and other uses.^40^ Before 2015, only about 20% of wastewater worldwide received proper treatment. A 2016 United Nations report states that nearly 70% of industrial wastewater in poor countries is discharged into the environment without proper disposal.^41^ In many underdeveloped nations, wastewater is still released into water bodies without adequate treatment.^22^Table 1 shows the different types of water contaminants, their sources, and the harmful effects they cause. Throughout history, people have used science-based techniques to purify polluted water, both in the past and the present day.^7^ Ongoing progress aims to develop new ways to treat wastewater to meet the demand for clean water.^42^ However, effectively treating wastewater, including contaminants, remains challenging with current methods.^43^ The study outlines various wastewater treatment techniques, which typically include biological, chemical, and physical methods organized into stages called preliminary, primary, secondary, and tertiary treatment.^44,45^ Each approach has specific advantages and limitations. Industrial applications often avoid methods that are costly to install and operate, time-consuming, produce limited output, or generate harmful byproducts, as shown in Table 2.^46^ Since it is responsibility of water providers to deliver and pure water, treatment methods must be characterized by long-term durability, reliability, accuracy, safety, and affordability.^40,47^ As a result, discovering an alternative treatment technique that can completely decompose or eliminate pollutants is essential.^26^

**Table 1:** Various types of water pollution, including their origins and impacts^39,40,48^

Types	Origins	Impacts
Organic	Pest control substances include insecticides, herbicides, and detergents	Issues with aquatic life
		Diarrhea, kidney damage, colon inflammation, hemolytic uremic syndrome, illnesses such as typhoid fever and salmonellosis, and Legionnaires' disease
Inorganic	Trace substances, metallic compounds, inorganic salts, heavy metals, and acidic minerals	Aquatic plant and animal concerns as well as problems with public health
		For example, heavy metals enhance the body's vulnerability to infections, alter the production and use of neurotransmitters inside the body, and produce reactive oxygen species, which may lead to oxidative stress
Nutrients	Fertilizers and plant detritus	Eutrophication is influenced
Contaminated water and sewage	Residential wastewater	Impact on the process of eutrophication
Industrial	Pollution originating from municipal areas	Induced water and air contamination
Agricultural	Water-soluble compounds containing nitrate and phosphorus ions	The fast development of algae leads to the depletion of water oxygen levels, resulting in fish death
Macroscopic	Oceanic waste	The accumulation of plastic waste causes environmental contamination
Radioactive	Multiple isotopes	Certain types of cancer, congenital abnormalities, and hereditary conditions
Suspended solids and sediments	Earth cultivation, destruction, and excavation operations	The process of fish reproduction is adversely affected, hurting the aquatic ecosystem, including insects and fish
Pathogens	Microorganisms such as bacteria and viruses	Water-related illnesses

**Table 2:** Some regular water and wastewater treatment methods, their relative performances and limitations

Strategies	Performance and drawbacks	Ref.
Bioremediation	Biological treatment is a commonly used technique for removing organic pollutants from water, although it is ineffective in eradicating halogenated organics and some organic halides. Furthermore, it is inefficient in eliminating endocrine-disrupting drugs and primary care pharmacist practitioners. When used in conjunction with filtration, it may be very successful in eradicating harmful materials. Nevertheless, it relies on microorganisms and may be affected by water content, loading rate, medium type, temperature, and aeration level. Over time, the performance of the technology might be diminished due to fouling and filter clogging	45, 49 and 50
Coagulation/precipitation	Chemical precipitation is a simple and effective process for eliminating harmful materials from the environment, but it is expensive due to the large quantities of chemicals required. Though not employed for organic pollutants, coagulation may remove colors from wastewater. After coagulation and flocculation, sedimentation or filtration may remove toxic and inorganic components from sewage. The high cost of chemical reagents makes coagulation and precipitation costly. The system must be carefully regulated and inspected to ensure the correct amounts of chemicals is introduced to clean the system without excess chemicals. Multiple metal species may complicate treatment owing to amphoteric compounds; optimizing one species' elimination might inhibit another. The effluent typically needs pH modification after treatment, increasing treatment costs. Coagulation and precipitation create a lot of waste, which is dangerous and expensive to treat due to the poisonous elements	40, 45, 49, 50 and 51
Chemical oxidation	Chemical oxidation is a technique used to address organic contaminants; however, it is not as efficient when dealing with hazardous components, inorganic pollutants, dissolved minerals, and salts. The inefficiency is often caused by the low concentrations of contaminants in the effluent. Chlorine is a toxic substance that is both corrosive and poisonous and generates harmful byproducts. Ozone is costly because of its elevated toxicity and the pollutants it produces. The manufacture and shipment of concentrated hydrogen peroxide can incur significant energy expenses. Wet oxidation necessitates elevated pressures and temperatures, leading to substantial costs. Chemical oxidation poses risks and necessitates cautious management of reactive chemicals and sludges. Chemical synthesis requires a significant amount of energy and necessitates frequent replacement of electrodes due to corrosion. Electrochemical oxidation is not practical in removing persistent contaminants from highly concentrated effluent	40, 45 and 50
Photocatalysis	Utilizing several photolysis methods, such as UV photolysis or TiO_2_-catalyzed UV photolysis, may be very efficient in breaking down a wide range of halogenated chemical compounds, some non-halogenated organic compounds, heavy metals, and particular personal care products. Photolysis alone may struggle to destroy volatile organic compounds at low concentrations, necessitating additional treatment methods. The effectiveness of photolysis is typically limited by the transparency of the water being purified, since UV light has to be capable of passing through it. The substantial energy consumption of UV photolysis may result in a significant life-cycle effect. UV lights need regular cleaning, and their replacement results in higher labor expenses. Photocatalysis is often ineffective over extended periods due to water chemistry factors, such as the presence of co-contaminants and hardness	40, 45, 47, 50 and 52
Filtration and membrane filtration	Various filtering methods are very efficient in eliminating most pollutants from wastewater. Filtration operates by capturing impurities inside the small openings of the filter, making it a very size-dependent method of elimination. Conventional filtering techniques are often insufficient for eliminating heavy metal ions, emerging pollutants, and other dissolved ions from water. However, membrane filtration, ultrafiltration, and reverse osmosis are all relatively efficient in addressing these issues	40, 45, 47, 49, 50 and 51
	Water movement across the membrane depends on applying pressure to push the fluid over the membrane, which may demand substantial energy inputs. In membrane filtration, particular circumstances must be maintained to avoid fouling, which is a typical problem in filtering. Backwashing is often necessary to prevent blockages, and the membrane is typically cleaned with strong chemicals to prepare it for regeneration. Reverse osmosis also necessitates adding minerals to the water and modifying the pH level, which incur additional expenses	
Adsorption	Most adsorption techniques use a carbonaceous substance to ensnare pollution particles inside its porous framework. Although the basic components for activated carbon may be cheap, using non-sustainable energy sources to generate high-quality activated carbon has been shown to affect the ecosystem substantially	40, 45, 47, 49, 51 and 53
	Reactivation of the carbon substance is necessary for activated carbon remediation to eliminate the adsorbed organic molecules. Alternative adsorption media are sometimes costly to manufacture and have a limited lifetime for reactivation, which is similarly costly. While some cheaper alternatives have been discovered, they often lack the same level of efficiency and frequently experience pore blockage. Adsorption eliminates the pollutant without altering it, producing a hazardous waste stream that must be managed. If the adsorbent is not reactivated on-site, it must be treated as hazardous waste, necessitating particular disposal methods and incurring additional expenses	
Fenton/photo-Fenton	The Fenton treatment employs an iron catalyst and hydrogen peroxide to oxidize pollutants in wastewater. Integrating UV irradiation into the procedure may augment the rate of oxidant generation, thereby increasing the treatment's efficiency. Fenton and photography Fenton reactions are effective for degrading a wide range of compounds, including halogenated organics, insecticides, herbicides, non-halogenated organics, colors, and particular personal care products. Fenton and photography: the Fenton processes encounter similar constraints as hydrogen peroxide oxidation and UV photolysis: hydrogen peroxide production incurs substantial costs due to the energy required, and powering the UV lamps also demands considerable energy expenditure. Handling Fenton reagents involves dealing with very reactive substances	45 and 49
Ion exchange	Ion exchange primarily utilizes synthetic materials, namely ion exchangers, as sorbents to selectively adsorb precious ions. In the biological sector, the primary ion exchange method is the application of ion exchange resin. This resin extensively extracts organic acids, amino acids, antibiotics, and other tiny compounds. The ion exchange process has many benefits, including cost-effectiveness, minimal equipment requirements, straightforward operation, and little or no reliance on organic solvents. However, some limitations are associated with it, including a lengthy manufacturing cycle, occasional subpar product quality, and significant pH fluctuations throughout the operation. In addition, it is not always feasible to locate the appropriate resin	51
Boiling	In communities today, this practice is widespread. Boiling drinking water kills bacteria and viruses, but not chemical contaminants. Pathogens and biodegradable contaminants may be decontaminated using this approach. Filtering filthy or impure water before boiling improves its quality. Many creatures die in water boiling at around 100 °C. When water is warmed to 70–75 °C, microorganisms are unlikely to live longer than thirty minutes. One of the easiest water purification procedures without extra equipment is boiling. Water becomes less hard and salty when boiled. Along with its benefits, this technology has significant drawbacks, such as the need for fuel and the difficulty of purifying massive amounts of water	7
Solar disinfection	Solar disinfection is a common and cost-effective home approach. This technique is quite time-consuming since it involves exposing water held in transparent plastic or glass containers, or obvious plastic bags, to direct sunlight for extended periods to destroy the pathogenic microorganisms contained in the water. Occasionally, when the situation is overcast or during the winter, it may take several days for the water to be thoroughly disinfected using this method. The ability of this approach is determined by the intensity of the sun, the absorption of light, the initial concentration of bacteria, and the turbidity of the water. Although this procedure is cost-effective, its time requirements make it less desirable	7

## 3. Adsorption

Adsorption is an efficient, environmentally sustainable, and extensively used technique for eliminating contaminants from water and wastewater. This method has attracted considerable interest owing to its straightforwardness, economic efficiency, and adaptability in mitigating diverse pollutants, such as pesticides, heavy metals, pharmaceuticals, and dyes.^54,55^ Adsorption basically consists of the transferring of contaminants, termed adsorbates, from a liquid or gaseous phase to the surface of a solid substance defined as the adsorbent (solute). This transfer happens as a consequence of physical or chemical interactions between the adsorbates and the adsorbents, leading to the deposition of pollutants on the surface of the adsorbents.^56^

**Fig. 4:**
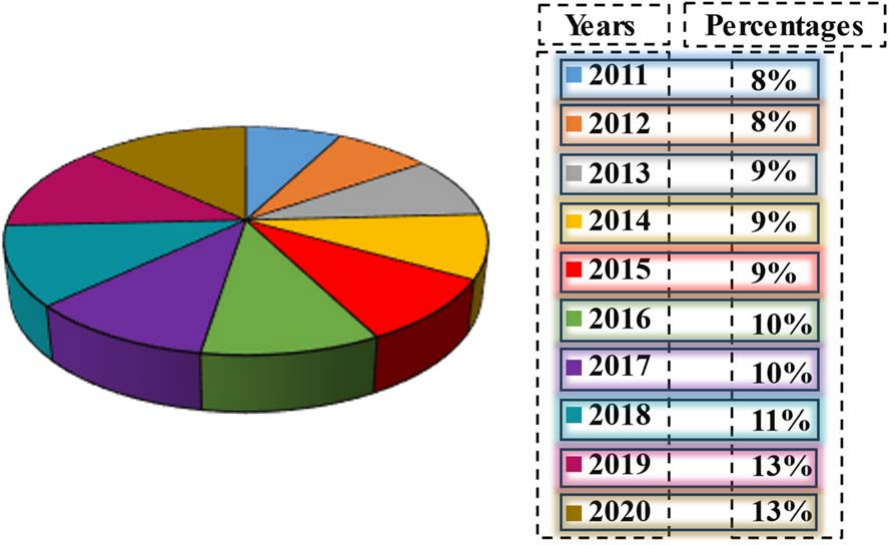
Increasing annual percentage of adsorption-related publications.^73^

The principle of adsorption has a substantial historical background. Preliminary research conducted by C. W. Scheele in 1773 illustrated the uptake of gases by charcoal, whilst Lowitz (1785) used charcoal to decolorize solutions of tartaric acid. Subsequently, Heinrich Kayser (1881) used the word “adsorption” to characterize the surface phenomena of material accumulation.^56^ These essential investigations established the basis for the extensive use of adsorption in water remediation applications. By the 20th century, practical applications, such as the removal of heavy metals by activated carbon with efficiencies over 99.9%, emphasized its promise for eliminating both organic and inorganic contaminants.^57^Fig. 4 demonstrates that articles related to adsorption are becoming more prevalent every year.

Adsorption is classified into two main categories: physical adsorption (physisorption) and chemical adsorption (chemisorption). Physisorption is regulated by weak van der Waals forces and hydrogen bonding, often resulting in the formation of monolayers or multilayers on the adsorbent surface. This reversible process needs minimum activation energy and is frequently preferred at low temperatures^58,59^. Chemisorption, by contrast, consists of intense chemical bonds, including covalent or ionic interactions, resulting in the establishment of a monolayer on the adsorbent. Chemisorption is often irreversible, highly selective, and characterized by a greater heat of adsorption, generally between 40 and 125 kJ mol^−1^.^60,61^ In optimal circumstances, physisorption and chemisorption may take place either simultaneously or in an ordered process. For instance, an adsorbate might first be physically adsorbed onto the surface and later establish chemical bonds.^56,62^ The distinction between physical and chemical adsorption is illustrated in Table 3. Covalent bonding and surface functionalization play pivotal roles in chemical adsorption processes. Functional groups such as carboxyl (–COOH), amino (–NH_2_), hydroxyl (–OH), and thiol (–SH) can form strong coordinate or covalent bonds with heavy metal ions, producing stable inner-sphere complexes that enhance adsorption selectivity and capacity. These groups act as electron donors to the vacant d-orbitals of metal ions, resulting in robust metal–ligand interactions. Furthermore, surface functionalization strategies significantly increase the density and accessibility of active sites while improving structural stability and regeneration potential.^63,64^

**Table 3:** A comparison of physical and chemical adsorption processes

Physical adsorption	Chemical adsorption
Physical adsorption is mainly influenced by weak intermolecular forces, including van der Waals forces, hydrogen bonding, and electrostatic interactions. It is a non-specific process, indicating that it could happen with various adsorbate–adsorbent combinations. Essential properties associated with this process consist of:	Chemical adsorption refers to the establishment of strong chemical bonds-either covalent or ionic-between the adsorbate and the adsorbent surface. This method is much more specific and selective than physisorption. The primary characteristics consist of:
• Small binding energy: the binding energy associated with physisorption is comparatively low, often between 5–40 kJ mol^−1^, representing small attractive forces.^56,62^ The binding energy has been reported as around 10–300 meV (ref. 58)	• Strong binding energy: distinguished by strong binding energies, often between 40 and 124 kJ mol^−1^, indicating powerful interactions. The energy scale may vary from 1 to 10 eV, based upon the nature of the chemical bond established^58,62^
• Reversibility: owing to the small interaction forces, physisorption is often reversible, facilitating the straightforward desorption of the adsorbate from the adsorbent surface (regeneration or reactivation)^74^	• Irreversible: due to strong bond formed, chemosorption is often irreversible. Desorption of chemisorbed species requires the rupture of these bonds, which makes it energetically challenging^70,74^
• Multilayer generation: may lead to the development of monolayers or multilayers of adsorbate molecules on the adsorbent surface^59^	• Monolayer generation: in contrast to physisorption, chemisorption is restricted to a monolayer owing to the specificity of chemical bonding^59^
• Non-activated technique: the method is characterized by low activation energy requirements, providing rapid operation^75^	• Activated technique: chemisorption needs activation energy, resulting in a slower method relative to physisorption^75^
• Temperature sensitivity: physisorption is generally enhanced at lower temperature levels. A rise in temperature often results in a reduction in adsorption capacity because the weak nature of the binding forces^65^	• Temperature independence: chemisorption could happen throughout an extensive range of temperatures, as its rate potentially improving with temperature due to the need of energy to activate^61^
• Mechanism of interaction: consist of van der Waals forces, hydrogen bonding, ion exchange, dipole–dipole interactions, π–π interactions, and hydrophobic hydration^56,57^	• Mechanism of interaction: the method includes transferring electrons or sharing, resulting in the creation of covalent or ionic bonds. The surface functional groups of the adsorbent substantially affect the chemisorption process^54,60^

The effectiveness of adsorption depends on various parameters such as the surface area, pore structure, and functional groups of the adsorbent, as well as the physicochemical characteristics of the adsorbate. External factors like pH, temperature, ionic strength, and contact time are also crucial^57,65^). For instance, cationic dyes typically rely on electrostatic attractions for adsorption, although hydrophobic or hydrogen bonding interactions may dominate in different systems.^56^ Surface modifications and adjustments, including functionalization with specific chemical groups, can enhance the selectivity and adsorption capacity of adsorbents.^66^

Generally, traditional adsorbents like activated carbon, zeolites, and silica gel have been widely used due to their high adsorption efficiency and stability. However, their high costs and regeneration challenges have spurred interest in alternate adsorbents derived from agricultural and industrial waste. Materials such as chitin, nutshells, rice husks, and fly ash offer sustainable and cost-effective options.^61,67^

Recently, nanoparticles have gained attention as innovative adsorbents because of their large surface areas, customizable surface chemistries, and rapid adsorption kinetics.^68^ Their performance and selectivity can be further enhanced by modifications such the adding of functional groups or surfactants.^65^

**Fig. 5:**
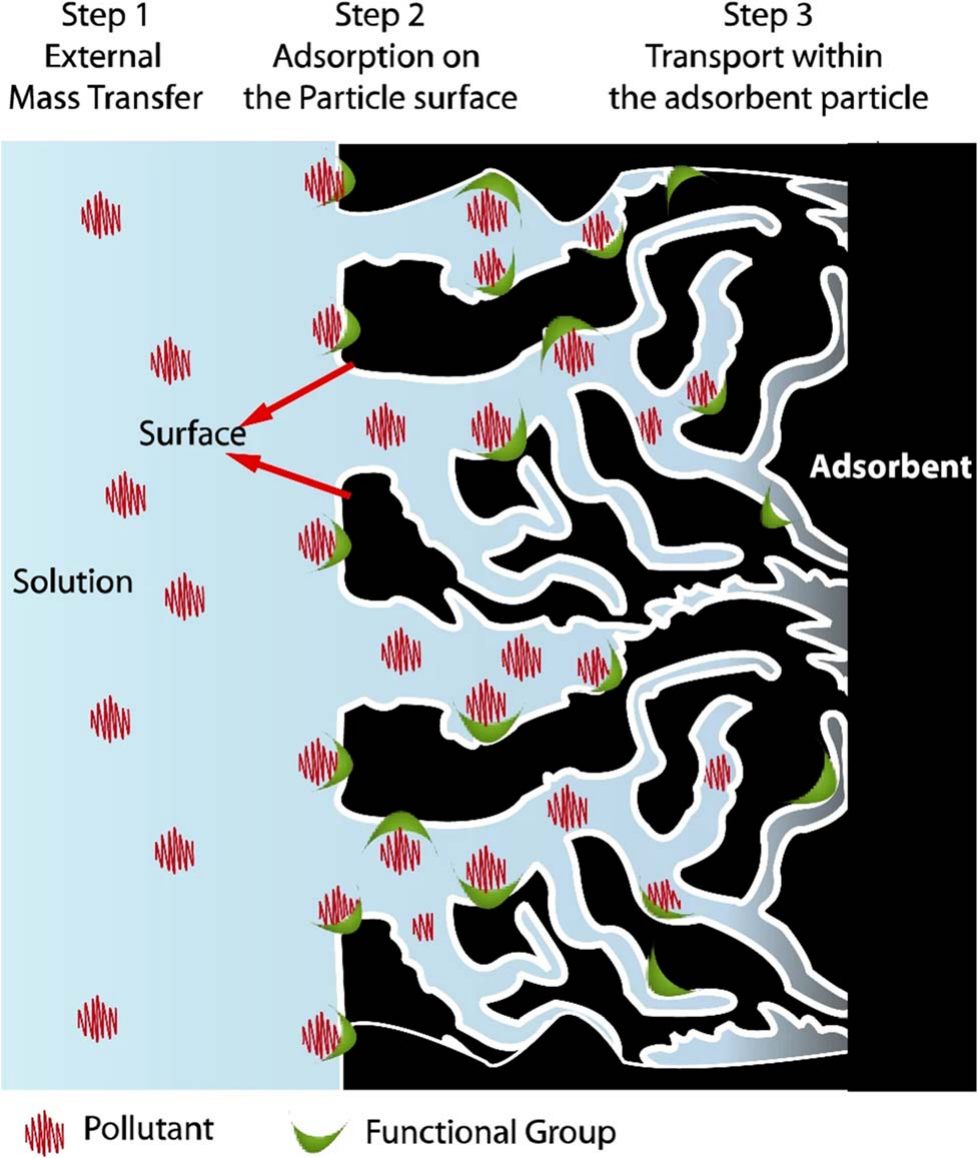
Schematic illustration of the adsorption technique and its mechanics. Adapted from ref. 57 with permission from Elsevier, copyright 2024.

The adsorption process generally proceeds through three stages; transportation of the adsorbate from the bulk solution to the surface of adsorbent, adsorption onto the surface, and diffusion inside the pores of the adsorbent.^54,57^ The stages are governed by diffusion, mass transfer, and thermodynamic principles aimed at minimizing the system's free energy. Interactions involved in adsorption include van der Waals forces, electrostatic attractions, hydrogen bonding, and chemical bonds, depending on the characteristics of the individual adsorbent and adsorbate. Fig. 5 illustrates these interactions, showing the sequential nature of the adsorption process.^57^

A key advantage of adsorption is its environmental sustainability. Unlike traditional technologies that produce hazardous byproducts or sludge, adsorption is a process that does not generate secondary pollution.^69^ Additionally, many adsorbents can be reactivated or regenerated through desorption, allowing multiple reuse cycles and reducing operational costs.^70^ This feature enhances the feasibility of adsorption for large-scale applications, especially in decentralized or resource-limited settings.

In conclusion, adsorption is a fundamental method for treating water and wastewater. Its ability to effectively remove a variety of pollutants, combined with its cost-effectiveness and eco-friendliness, underscores its importance in addressing global water pollution. Ongoing improvements in adsorbent materials and optimization techniques are expected to enhance the efficiency and sustainability of adsorption systems, ensuring their continued role in sustainable water management.^71,72^

## 4. Nanotechnology

**Fig. 6:**
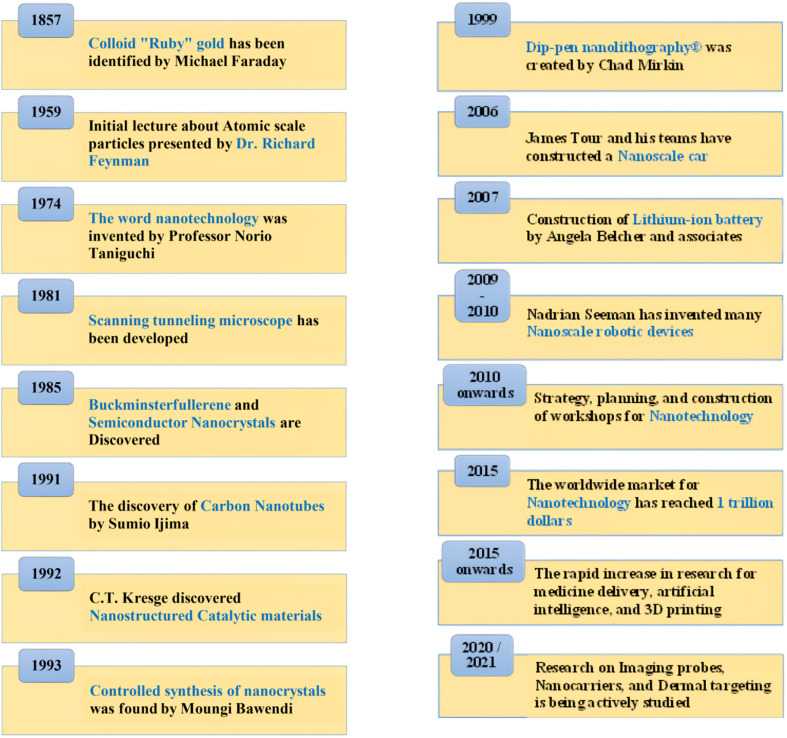
Progression of events in the area of nanotechnology across time.^78^

The term “Nano” originates from the Greek word “nanos,” which refers to “dwarf.” A nanometer is a unit of length equivalent to one billionth (10^−9^) of a meter, about the length of 10 hydrogen atoms. The width of an average human hair is around 80 000 nanometers. Nobel Laureate Professor Richard Feynman introduced the principle of nanotechnology in his renowned 1959 speech titled “There is plenty of room at the bottom”.^76^ Nanotechnology integrates engineering capabilities with science at the atomic and molecular scale.^77^Fig. 6 shows the progression of nanotechnology, with notable research advancements beginning around 2010 and a sharp increase from 2015 forward.

**Fig. 7:**
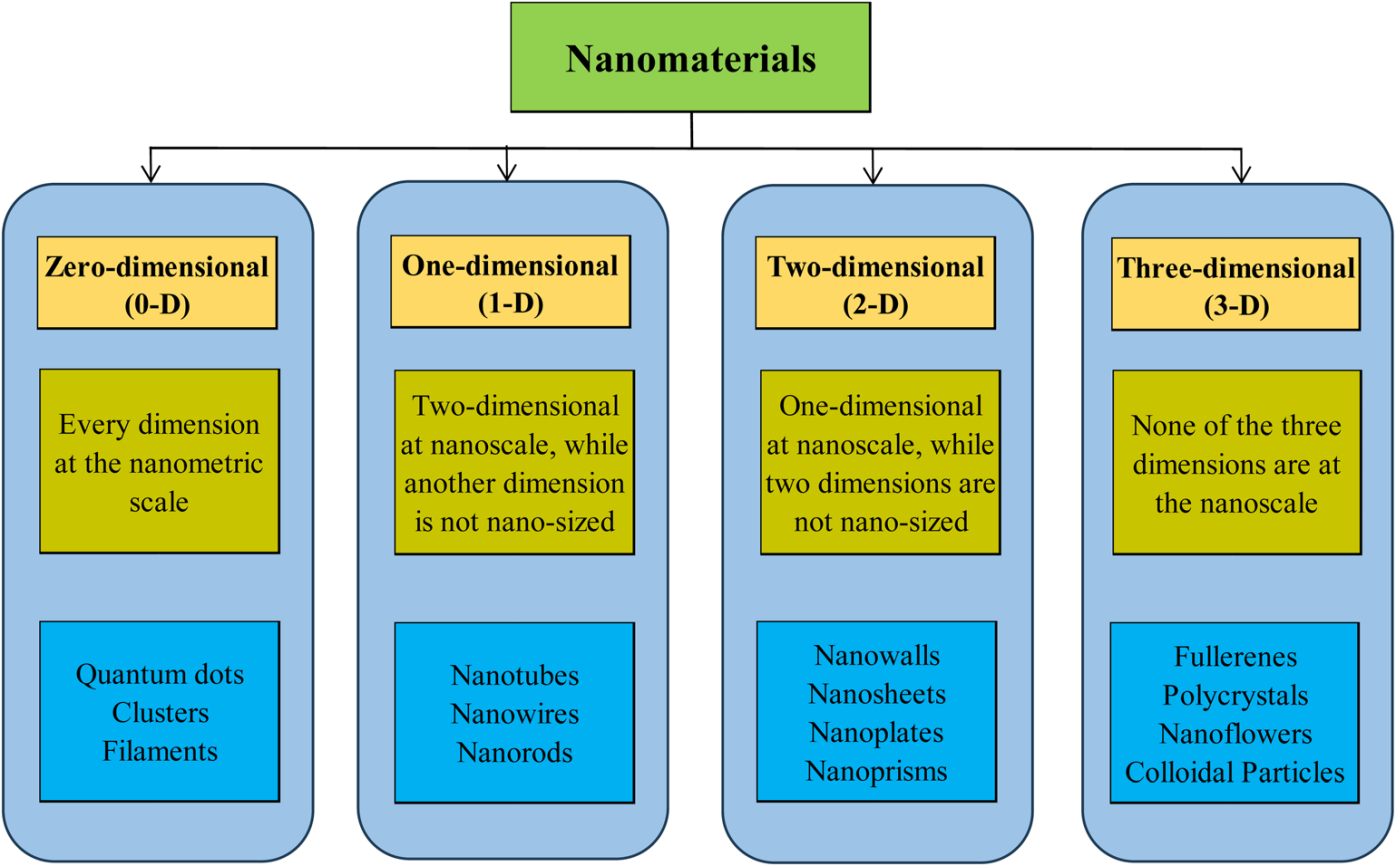
Various categories of nanomaterials based on architectural configuration.^80,83–88^

**Fig. 8:**
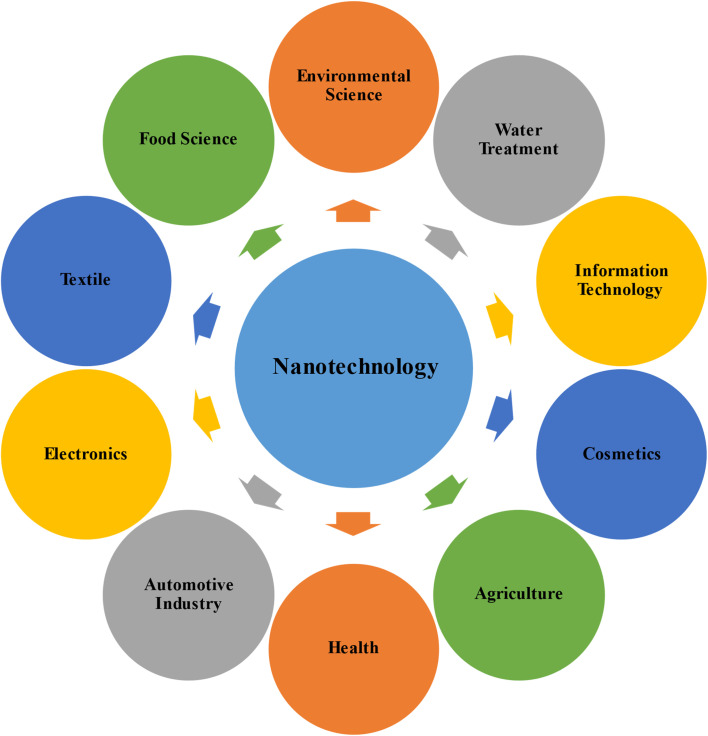
Schematic illustration of the wide-ranging applications of nanotechnology across various fields.^82,83^

Nanotechnology is the application of phenomena at the nanoscale size, which is the discipline of nanoscience in general. Nano-materials are the finest elements created by mankind, measuring only a few nanometers in dimension.^79^ According to the European Commission, a nanomaterial is a microscopic particle, originating naturally, produced incidentally, or deliberately fabricated. These particles can exist individually (unbound) or together in agglomerates. For a material to qualify as a nanomaterial, it must contain at least half of its particles within the size range of 1–100 nanometers.^80^ A wide range of nano-materials have been created, including nanotubes, nanowires, particles, films, colloids, and quantum dots.^52^ Nanomaterials could be categorized based on their architectural configuration, which influences their properties and applications, as demonstrated in Fig. 7. This innovative subject incorporates knowledge and principles from multiple fields, such as physics, biology, chemistry, and biotechnology.^81^ Nanomaterials possess extraordinary characteristics due to their very minuscule dimensions, which provide an extensive range of potential applications in several areas.^82^Fig. 8 illustrates the potential applications of nanotechnology in several fields.

**Fig. 9:**
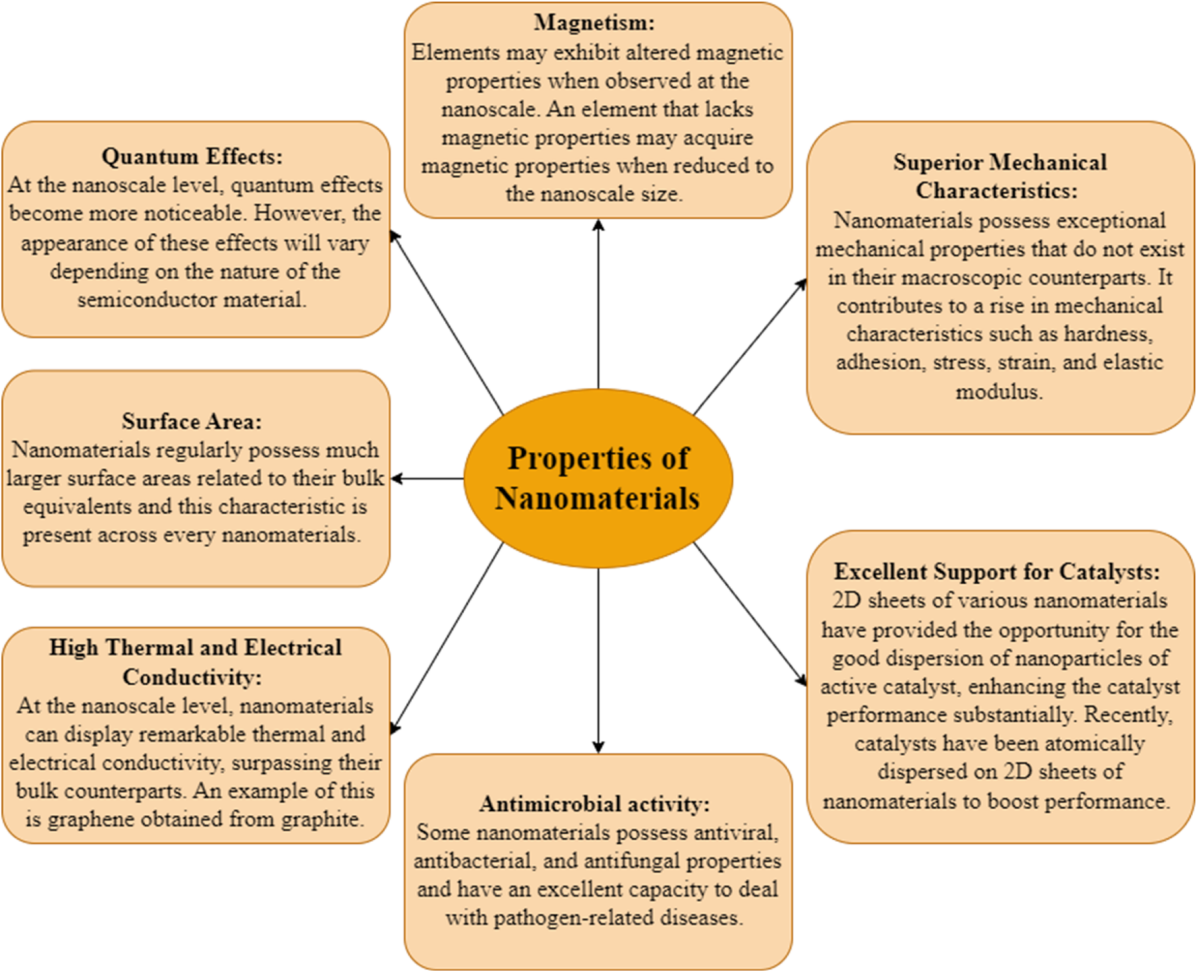
Exploring the characteristics of materials at the nanoscale.^76,91,92^

**Fig. 10:**
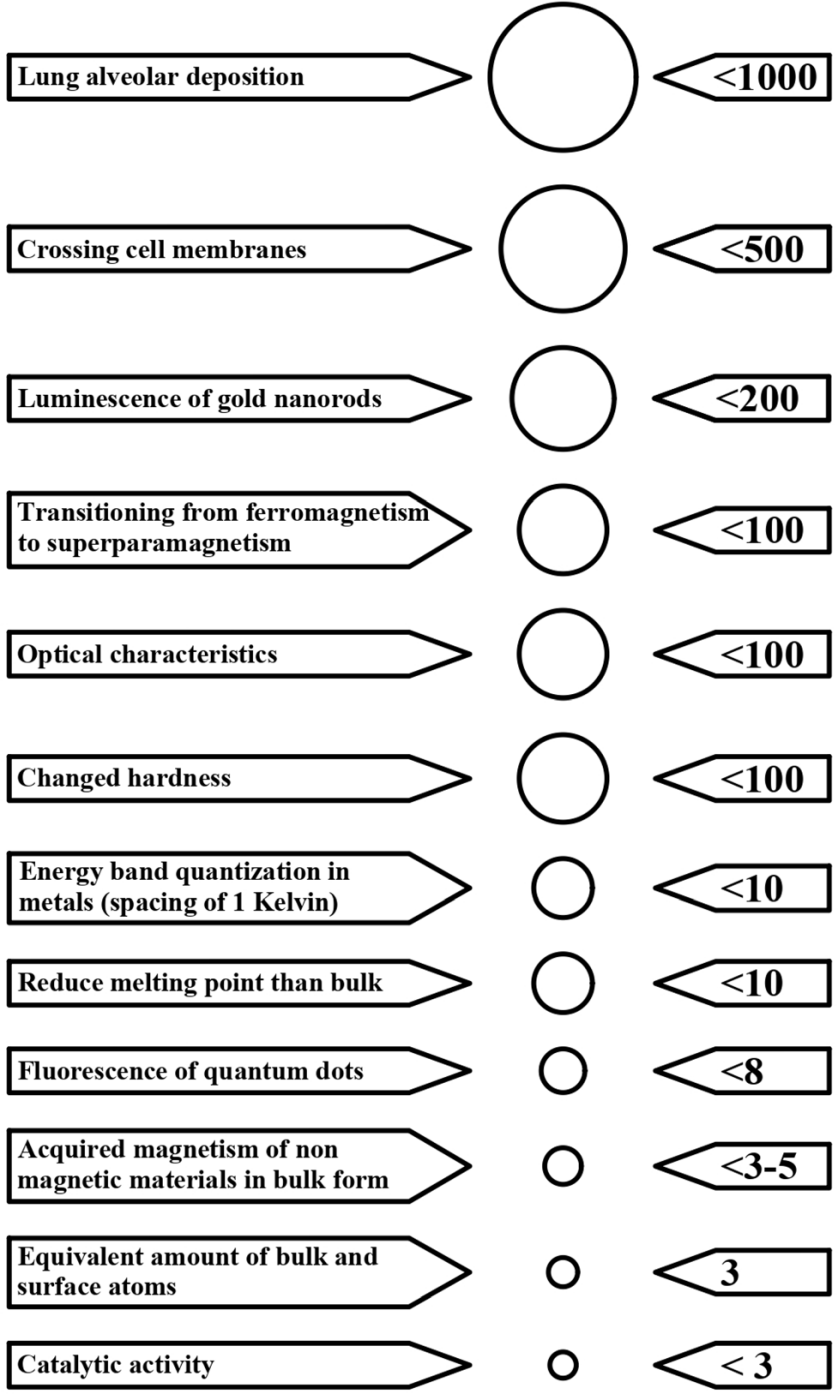
Nanoparticles experience alterations in chemical, physical, and mechanical characteristics at certain dimension thresholds, distinguishing them from larger particles and bulk materials.^93–97^ The units used for measurement are nanometers, and the picture is not drawn to scale. The change may occur gradually, as indicated by the melting temperature, or rapidly, as observed in catalytic activity. Surface or quantum effects could cause the difference.

Substances have distinct and highly modified characteristics when scaled down to the nanoscale (as shown in Fig. 9), making them suitable for a diverse array of uses in the remediation of wastewater.^89^ The characteristics and functions of materials may change when they are modified from a large scale to a nanostructured scale.^90^ The dimensions, while particular characteristics vary about the bulk of the equivalent material, are shown in Fig. 10.

### 4.1 Application of nanotechnology in wastewater

Nanotechnology has emerged as a significant field in environmental remediation, providing novel approaches for handling essential issues in wastewater treatment.^98^ Presently, several sectors worldwide, including paper and pulp, textiles, petroleum and natural gas, and pharmaceuticals, utilize substantial quantities of water and therefore generate heavily polluted wastewater.^99^ The use of nanoparticles may provide prospects for the effective remediation of heavily polluted wastewater.^100^ The hardest pollutants to remove from wastewater exist in the nanoscale region, namely between 1 and 100 nanometers. Therefore, nano-based methods are particularly appropriate for addressing these pollutants. Nanotechnologies provide significant benefits for water decontamination due to their small size and the exceptional physicochemical characteristics of nanoparticles.^6^ Their increased surface area-to-volume ratio and surface reactive properties create a high density of active sites for contaminant interaction, facilitating exceptional adsorption efficiency. Nanomaterials demonstrate significant efficacy in eliminating pollutants from aqueous environments.^101^

The diminutive dimensions and distinctive surface properties of nanomaterials enhance their interactions with pollutants *via* mechanisms including van der Waals forces, hydrogen bonding, and electrostatic interactions. Moreover, techniques for surface functionalization enable the customization of nanomaterials for the targeted adsorption of particular pollutants. Functionalized nanomaterials have been effectively utilized to target heavy metals, dyes, and pharmaceuticals, showcasing their versatility and adaptability in various wastewater treatment contexts.^58,102^ Furthermore, specific nanomaterials like titanium dioxide and zinc oxide demonstrate photocatalytic characteristics, producing reactive oxygen species when exposed to light, which facilitates the degradation of pollutants. This multiple capability significantly improves their capacity for comprehensive wastewater treatment.^101^

Many efficient, environmentally friendly, and economical nanomaterials can be used to treat water and wastewater. These nanomaterials have distinct physical, chemical, and biological properties, giving them unique capabilities for decontaminating industrial effluents, drinking water, surface water, and ground water. The materials may be classified as organic, inorganic, carbon-based, or composites.^52,103^ Implementing nanotechnology in the purification and reusing of household water presents potential solutions to issues associated with deteriorating water quality in network distribution, less reliance on critical infrastructure, and the utilization of alternative water sources for farming and portable purposes while subsidizing energy use. This approach is particularly relevant for developing countries facing challenges related to decreasing quality of water and increasing demand for purer water to ensure food safety, healthcare, and compliance with environmental regulations.^11^

**Fig. 11:**
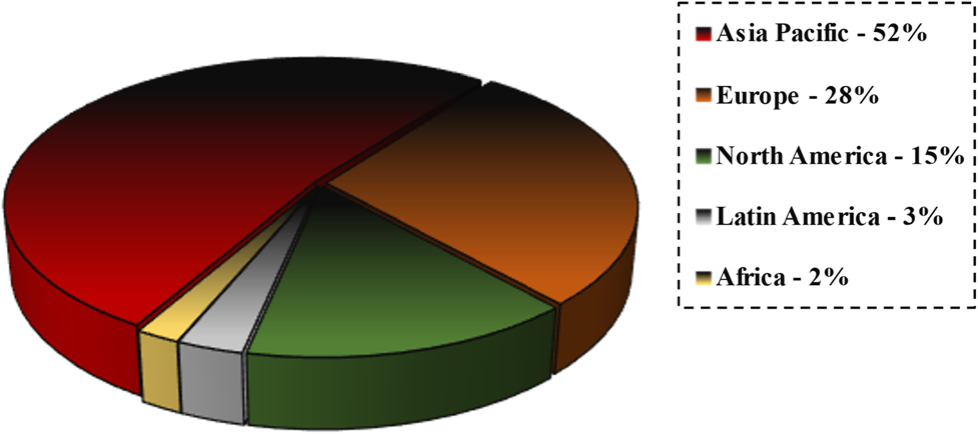
Global nanotechnology market study report (2020), illustrating the statistics of the utilization of nanotechnology-based products worldwide.^104^

Governments and commercial businesses throughout the world, especially in China, Japan, the United States of America, and Germany, have initiated some programs and attempts to further the development of nanotechnology.^40^ Many experts have been conducting different research and experiments on the potential applications of nanotechnology in water and wastewater remediation. However, there has been little advancement in comparison to the implementation of nanotechnology in areas like medicine and electronics.^45^ Research using nanomaterials for water and wastewater treatment began in the early 2000s, but was mostly focused on laboratory and pilot-scale experiments. China and Iran were recognized as the leading nations in terms of the number of publications on this scientific subject.^99^ As shown in Fig. 11, the 2020 market research of nanotechnology indicates that the Asia-Pacific region is the most appealing market for nanotechnology, due to significant demand for prospective nanomaterials.^104^

Nanomaterials exist in several forms, such as nanoparticles, nanotubes, nanofibers, and nanosheets, each specialized for particular functions. Carbon nanotubes coated with titanium dioxide have been modified to improve adsorption effectiveness owing to their increased surface area and better reactivity. Smaller hematite nanoparticles have enhanced adsorption capabilities for metal ions at reduced pH levels, highlighting the influence of particle size and shape in adsorption efficacy.^57,98^ Advanced synthesis methods, including sol–gel techniques and molecular self-assembly, have enhanced the performance and scalability of nanomaterials for industrial applications.^57^

Nanomaterials, such as nanoparticles, nanotubes, and nanomembranes, are capable of detecting and removing various biological and chemical particles, including toxic elements of heavy metals, dyes, organic substances, bacteria, viruses, antibiotics, micronutrients, and algae.^105,106^ Previous research has consistently demonstrated that nanomaterials possess essential abilities and suitability for use in water and wastewater treatment. Specifically, they have been proven effective in membrane techniques, adsorption, catalytic oxidation, disinfection, and detection and monitoring. Unfortunately, the majority of the established nanomaterials were still in the laboratory investigation phase or were only serving as a demonstration of feasibility. A specific type of nanotechnology widely accessible through injection is zero-valent iron nanoparticles.^41,107^ The United States commonly utilizes zero-valent iron nanoparticles for groundwater remediation. Due to the lower costs of nanomaterials, they are economically sustainable for water and wastewater treatment, making them a more acceptable option. Nevertheless, basic treatment limitations are associated with directly employing free nanoparticles in water and wastewater treatment operations. Nanoparticles tend to accumulate together in fluidized systems or fixed beds, which leads to a significant decrease in activities and a decrease in pressure.^108^ Additionally, except for magnetic nanoparticles, separating the majority of exhausted nanoparticles from the purified water for subsequent reuse remains a difficult effort.^109,110^ Furthermore, much is still to learn about how nanomaterials behave and what happens during water and wastewater treatment. Understanding the effects of nanomaterials on the environment and human health is crucial, as it poses a significant challenge to the widespread use of nanotechnology.^111,112^

To prevent or reduce the possible negative impact caused by nanotechnology, creating a substance or instrument that may decrease the escape or movement of nanoparticles while still protecting their strong reactive properties can be preferable. The efficiency and potential of nanocomposite progress have been established. Nanocomposites are often produced by incorporating nanoparticles onto different types of supporting substances, such as polymers or membranes.^107^ Several nanocomposites that were documented had exceptional efficacy in purifying water, were capable of being reused, had a reasonable cost, and were suitable with current infrastructure.^108,113^ Recent improvements in the integration of nanomaterials with other substances have enhanced their adsorption effectiveness and decreased manufacturing costs, hence increasing their accessibility for large-scale applications.^114^

In the last ten years, nanoremediation has been extensively used for the treatment of polluted locations, displaying considerable benefits compared to traditional treatment techniques. Reports from the USEPA and environmental nanotechnology sources demonstrate that nanoremediation has resulted in an estimated 80% reduction in operating expenses and a significant drop in the duration necessary for site decontamination. The worldwide investment in nano-enabled technologies has significantly escalated from 432 million dollars in 1997 to 4.1 billion dollars in 2005, illustrating the increasing importance of nanotechnology across several fields. The market for nanotechnology-based products is growing quickly, with around 3 to 4 innovative products launched weekly. In 2015, the Asian nanotechnology industry was valued at around 14 741.6 million dollars, with forecasts predicting an increase to 55 056 million dollars by 2022, propelled by a compound annual growth rate of 20.7%. Market assessments from 2020 indicate that the Asia-Pacific area is the most appealing market for nanotechnology, driven by the rising demand for sophisticated nanomaterials (Fig. 11).^104^ The application of nanoparticles in water and wastewater treatment has shown notable increase. A study investigating *via* Scopus revealed 1990 papers relating to “nanomaterials in wastewater treatment”, indicating a growing scientific interest in this area of research.^38^

**Fig. 12:**
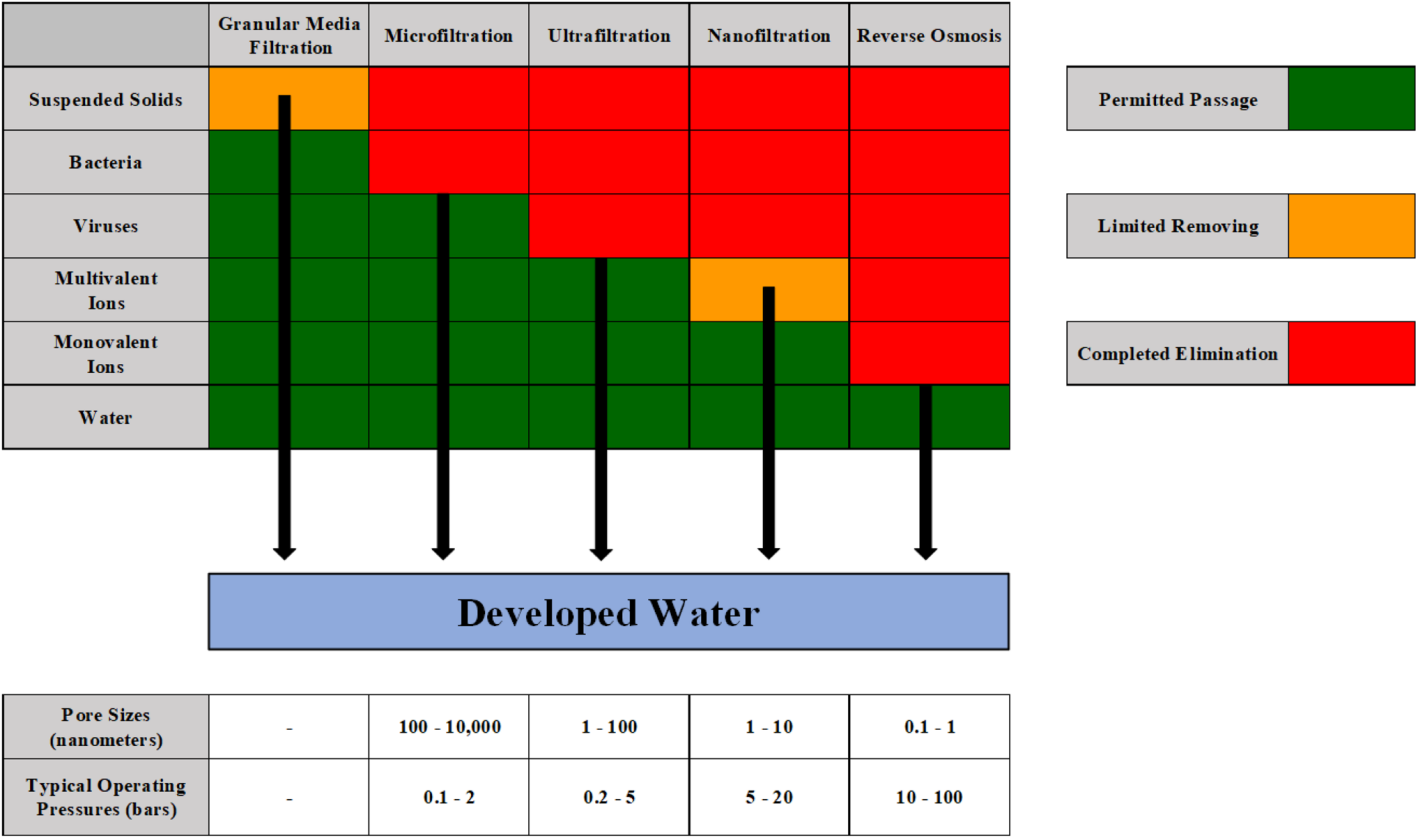
The effectiveness of various methods of filtration in removing contaminants.^7,85,121,122^

Nanotechnology's significant applications in wastewater treatment can be broadly categorized into three distinct groups according to the characteristics of its nanomaterials: nanomembranes, nanoadsorbents, and nanocatalysts.^52,68^ Nanomembranes are a crucial category of nanomaterials employed in wastewater treatment operations. Nanomembranes may be categorized into several types based on their porosity, structure, and application method.^115,116^ In this innovation, the pressure-driven use of wastewater has been demonstrated to be optimal for enhancing the desired water quality. Nano-filtration is widely used in industries for wastewater treatment due to its small pore sizes, cost-effectiveness, excellent effectiveness, and ease of use.^52^ Nanomembranes could be created using various nanomaterials, including nanometal particles and nano-carbon tubes, among others.^117^ The advantages of this technology are high-quality purified water, efficient disinfection, and minimal plant area requirements, the primary drivers behind its development. Furthermore, in comparison with alternative remediation methods, it is very affordable, effective, and straightforward in construction.^118,119^ The water has undergone advanced filtration procedures, offering superior-grade potable water. Enhanced purifying techniques include microfiltration, ultrafiltration, nanofiltration, reverse osmosis, and advanced oxidation. These membranes integrate adsorption, size-selective filtration, and catalytic functionality to efficiently eliminate pollutants like pharmaceuticals, heavy metals, and salts.^120^Fig. 12 illustrates the efficacy of several filtering methods in eliminating various pollutants often found in water.

The next category of nanomaterials is adsorption. Current research in nano-adsorption technology has examined chiefly the efficiency of using nano-adsorbent materials to eliminate contaminants from wastewater.^52^ Chemically active components with a high adsorption capacity could be employed to generate nanoadsorbents that attach to the outside of nano-materials.^123^ Their increased porosity, modifiable surface chemistry, and active surface sites render them very efficient in adsorbing a diverse array of pollutants, including trace metals and polar organic molecules.^62^ Various materials are used to produce nanoadsorbents, such as activated carbon, metal oxides, clay materials, silica, and modified materials in the form of composites.^117^ Nanoadsorbents have two primary characteristics: inherent surface and outside functionality. Their outside structure, visible size, and inner composition also influence their chemical, physical, and material characteristics. In an aqueous environment, the factors that impact the adsorption technique include a large surface area, strong adsorption activity, considerable chemical activity, atom arrangement on the surface, minimal inner diffusion resistance, and considerable surface binding energy.^52,124^ The most extensively investigated approach for eliminating contaminants from water and wastewater is the utilization of engineered nano adsorbents.^99^

Nanocatalysts are the final classification of nanomaterials. Researchers are interested in developing wastewater treatment innovations, particularly in using inorganic nanomaterials like metal oxides and semiconductors. Their elevated surface area-to-volume ratio improves catalytic efficiency, allowing for the breakdown of contaminants by mechanisms like reducing activation energy and promoting selective chemical reactions. A variety of nanocatalysts are used to break down pollutants in wastewater. These include electrocatalysts, Fenton-based catalysts, photocatalysts, and catalysts with antimicrobial characteristics. These catalysts contribute to enhancing the oxidation process of organic contaminants and have antimicrobial effects.^68,79,125–127^Table 4 provides a concise overview of the practical considerations regarding using nanomaterials to eliminate environmental pollutants, allowing for a brief evaluation.

**Table 4:** The practical considerations regarding the use of nanomaterials for effectively eliminating pollutants from the water and wastewater

Nanomaterials	Contaminants	Advantages	Disadvantages	References
Nano-membrane	Salts	Flexible design	Significant energy requirements	22, 40, 129 and 130
	Organic	Non-toxic	Membrane fouling and clogging	
	Contaminants	Environmentally sustainable	Extensive utilization of pressure	
	Inorganic	Excellent water quality	Expensive	
	Contaminants	Reduced establishment area	Expensive maintenance costs	
	Microbes	Cost-effective without losing efficiency compared to other new and traditional methods	The performance technique is relatively slow	
		
Titanium dioxide (TiO_2_) based	Heavy metals	Cost-effective	Low absorption capacity for both visible and ultraviolet radiation	131
	Dyes	Simple to prepare	The challenge of extracting TiO_2_ particles	
	Pesticides	Non-toxic		
	Microbes	Biocompatibility	Tend to clump together and form larger particles	
	Hormones and other endocrine-disrupting compounds	Strong chemical and corrosion resistance		
Dendrimer	Organic contaminants	Strong adhesion with little formation of sediment	Expensive	40
	Heavy metals	Regenerative		
		Simple separation		
Iron-based	Organic contaminants	Inexpensive	Generation of sludge	40, 132 and 133
	Heavy metals	Abundant in nature	Challenging removal of sludge	
	Dichlorinations	Uncomplicated preparation	Health concern	
	Dyes	Safe for handling	Challenging to recover	
	Anion and oxyanion	Characteristics of superparamagnetic		
		Groundwater purification		
		High adsorption efficiency		
Micelle	Organic	On-site remediation	Expensive	40 and 134
	Contaminants	Remarkable attraction towards hydrophobic		
	Dyes			
	Pharmaceuticals			
	Pesticides			
	Personal care products			
Bimetallic	Organic contaminants	Enhanced reactivity	Generation of sludge	22, 40, 135 and 136
	Inorganic metals		Difficult for recovery	
	Heavy metals		Challenging removal of sludge	
	Pharmaceuticals			
	Dichlorinations			
	Dyes			
	Pathogenic microorganisms and biotoxins			
	
	Radionuclides			
Nano-clay	Dyes	Non-toxic	Impermeable	40, 137 and 138
	Anion	Cost-effective	Generation of sludge	
	Heavy metals	Abundant in nature		
	Pharmaceuticals	Enhanced ion-exchange capacity		
	Organic contaminants	Increased efficiency of adsorption		
		Swelling potential		
		Excellent thermal characteristics		
		Biodegradable		
Magnetic	Organic	Low aggregation	Adverse environmental impacts and human health effects	40, 78 and 139
	Contaminants	Durability		
	Pharmaceuticals	No generation of sludge		
	Pathogenic microorganisms and biotoxins	A significant level of reusability and ability to regenerate		
	
	Dyes			
	Heavy metals			
	Personal care products			
Carbon nano-tube	Organic	Excellent chemical durability	Adverse environmental impacts and human health effects	22, 40 and 140
	Contaminants	Strong mechanical characteristics	Lesser adsorption efficacy	
	Heavy metals	Special electrical characteristics	Production expenses	
	Inorganic		Generation of sludge	
	Contaminants		Difficult for recovery	
	Anion			
	Aromatic			
	Contaminant			

A significant benefit of nanomaterials is their capacity for regeneration and reuse, which improves sustainability and lowers operating expenses. The capacity to recover and reutilize nanoadsorbents, for example, corresponds with sustainable practices and mitigates issues related to resource consumption and waste production.^128^ The progress in nanotechnology presents several possibilities for developing cost-effective and eco-friendly water supply methods. Nanomaterials are anticipated to possess diverse characteristics that provide cost-efficient and highly efficient methods for treating wastewater and water. These techniques would depend less on complex infrastructure for wastewater treatment and could be used with other water treatment processes such as adsorption, coagulation, membrane technology, and photocatalysis.^11,27^ Nanotechnology-based water and wastewater treatments show great potential in addressing the primary obstacles in water treatment and offer innovative treatment capabilities that can facilitate the cost-effective utilization of other water sources to enhance the water supply.

## 5. Characterization techniques

**Fig. 13:**
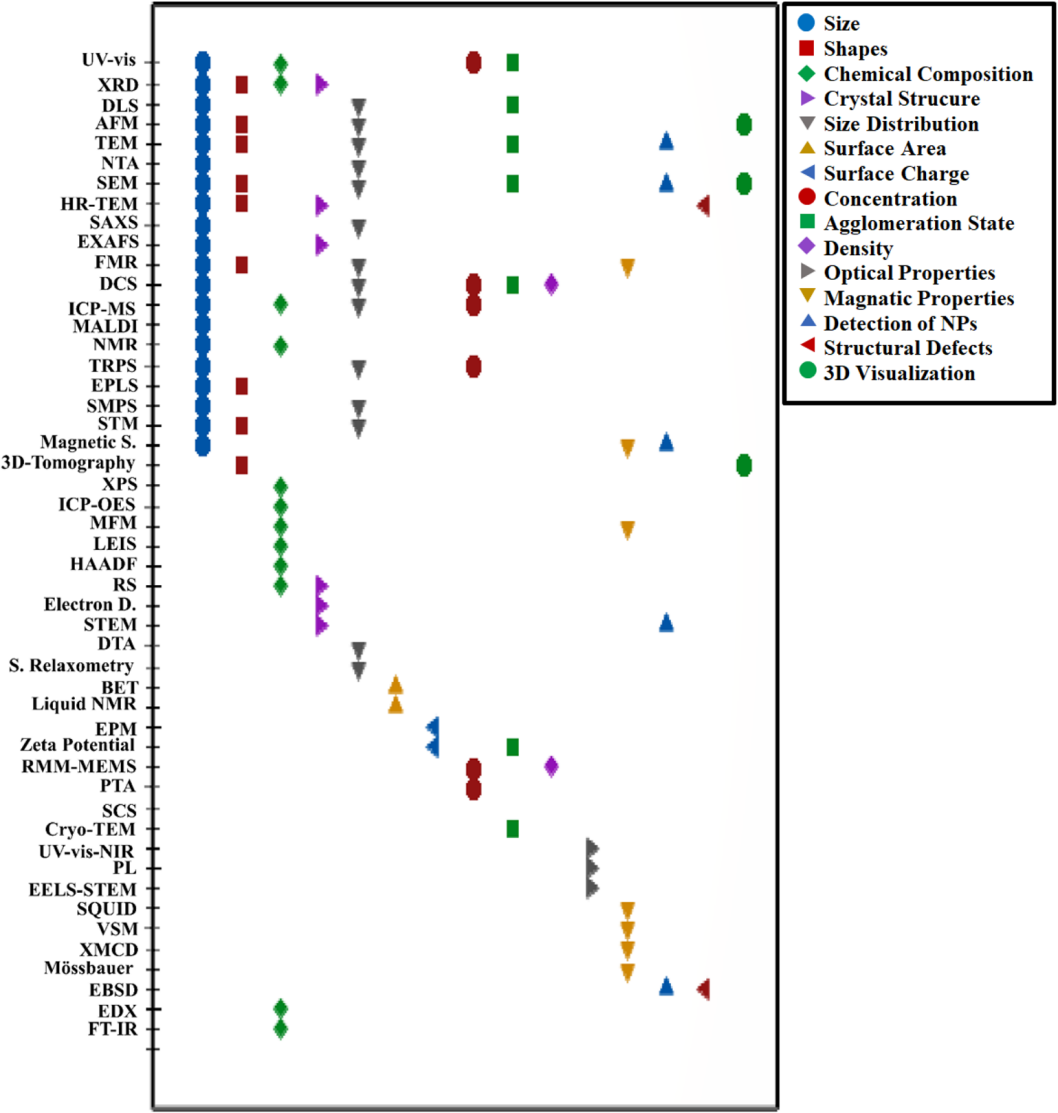
Several parameters and their corresponding methods of characterization (Magnetic S. = magnetic susceptibility; Electron D. = electron diffraction).^31,145,147,158,159^

Nanomaterials often exhibit distinct features compared to their larger counterparts, primarily due to their increased surface-to-volume ratio, significantly enhancing molecular-level reactivity. This results in unique electrical, optical, and chemical properties, and can affect their mechanical characteristics. Comprehensive characterization of nanoparticles is essential to understand and control their manufacturing processes fully. This includes analyzing agglomeration, spatial reasoning, geometric proportions, Brownian motion, intercalation, and dispersion, as well as particle size, crystal size, porosity, solubility, surface properties, water sorption, and surface morphology.^141,142^ Detailed and precise characterization of nanomaterials is vital to explore their enhanced properties, which allow overcoming limitations of conventional technologies. The methodologies for nanoparticle characterization are crucial for verifying the existence of nanoscale particles and assessing their quality.^81^ These approaches involve microscopy for visualizing samples and spectroscopy for analyzing their composition and structure. Characterization provides information on morphology, crystal structure, chemistry, electronic structure, mechanical, thermal, and electrical properties.^143^ Characterizing nanoparticles also helps determine their potential toxicity, stability, and dispersion behavior.^144^Table 5 presents various methodologies for nanoparticle characterization, while Fig. 13 displays parameters and their corresponding characterization methodologies.

**Table 5:** Some methodologies that can be used to characterize nanoparticles

Analysis method	Their roles in nanoparticle analysis	Ref.
X-ray diffractometry (XRD)	XRD is a technique used to analyze the crystalline structure of nanoparticles, providing information on crystallinity and chemical compound resolution. When a single-colored beam is aimed at the crystal, it generates distinct diffraction patterns, which are then analyzed using Bragg's equation to determine the features of crystalline or polycrystalline materials. The measurements are expressed in angstroms and are the main factor in determining a crystal's characteristic size and unit dimensions	146
Ultraviolet-visible spectrophotometer (UV-Vis)	UV-vis absorption spectroscopy is helpful in studying the absorbance bands and band gap of nanoparticles, particularly noble metals. It is used to validate nanoparticle formation and monitor stability. UV-visible spectroscopy is used to measure the optical properties of nanoparticle solutions, validate their synthesis, and assess their stability. It is a quick, sensitive, and user-friendly technique that can evaluate both solid and liquid samples, providing insights into optical properties, size, concentration, agglomeration state, and NP shape	31, 141, 142 and 147
Fourier transform infrared spectroscopy (FT-IR)	An FT-IR spectrophotometer is used to identify functional groups in synthesized nanoparticles, which are crucial for adsorption characteristics. It analyzes chemical functional groups between 400 and 4000 cm^−1^ in the spectral band, including carboxyl, hydroxyl, and amine groups. FT-IR spectroscopy is a rapid, cost-effective technique that requires minimal sample preparation. This technology collects detailed information with great precision over a broad spectrum, enabling the examination of the chemical composition of metal nanoparticles on surfaces and detecting organic, inorganic, and polymeric substances using infrared radiation	147, 148, 149 and 150
Scanning electron microscope (SEM)	SEM is a crucial analytical technique that uses electrons to generate micro-images, allowing for the detection and characterization of nanoparticles with varying sizes and shapes. It is particularly effective for determining the morphological structure and dimensions of nanoparticles	141, 147, 149 and 151
	SEM is a technique that uses an electron beam to analyze nanoparticle samples, providing detailed information on their surface morphology and chemical composition. This method examines various materials, including organic, inorganic, carbon-based, biological, and complex substances. The resulting images are used for morphological examination, allowing a comprehensive understanding of the material's physical structure	
Transmission electron microscopy (TEM)	TEM is a microscopic method to obtain detailed information on a material's surface features, structure, dimensions, composition, and crystallography. It involves the passage of an electron stream through a thin specimen, resulting in the interaction of electrons with the object and the formation of an image. TEM pictures precisely detail nanoparticles' specific dimensions, dispersion, and external structure. TEM provides higher-resolution pictures than a light microscope and is used for studying the structure and presence of nanoparticles	141, 142 and 148
Energy dispersive X-ray (EDX or EDS)	EDX is a crucial instrument in nanotechnology for determining the elemental composition of samples. It is an analytical technique used to chemically characterize or analyze the elemental composition of nanoparticles, providing data on chemical composition, phase identification, and purity. EDX is used to analyze nanoparticles to determine their elemental compositions, abundance, and purity. It is sensitive to surface chemistry and can detect elements in a very thin layer close to the surface. EDX can obtain compositional data on quasi-bulk specimens, and analyze single particles, morphologies, or isolated spots on filters or deposits. It is also compatible with applications like TEM and SEM	147 and 152
Thermogravimetric analysis (TGA)	TGA is a method for assessing a sample's chemical and physical characteristics by quantifying the reduction in mass as the temperature increases. It is often used in qualitative analysis to ascertain nanoparticle composition, evaluate additive impact, and gauge nanoparticle stability under oxidative and thermal conditions	149, 153 and 154
	TGA can also be used to conduct dehydration studies and study the reduction in material mass caused by oxidation, decay, or volatilization. This analysis generates a plot showing the relationship between temperature and mass	
X-ray photoelectron spectroscopy (XPS)	X-ray photoelectron spectroscopy (XPS) is a widely used for analyzing materials' elemental composition and chemical state. It determines electron binding energy by examining the kinetic energy of emitted photoelectrons and their corresponding wavelength in incoming X-rays. Plotting the intensity of photoelectrons against binding energy helps identify the chemical composition of materials and the oxidation states of each element. The variation in binding energy for an atom with the same oxidation state may indicate distinct local coordination environments. XPS is highly sensitive, allowing accurate analysis of components in small quantities, making it a popular method for measuring doped materials	155
Vibrating sample magnetometry (VSM)	Vibrating sample magnetometry (VSM) is a widely used technique for measuring hysteresis loops at room temperature, quantifying magnetic characteristics of substances based on magnetic field, temperature, and duration. It helps identify whether nanoparticles exhibit paramagnetism or diamagnetism, determining their classification as ferromagnetic or diamagnetic. A material's level of attraction to a magnet or magnetic field determines its classification, with paramagnetic materials not strongly attracted to a magnet, and diamagnetic materials repelling against a magnet after being magnetized	149, 153 and 156
Dynamic light scattering (DLS)	DLS is a technique used to study nanoparticle size distribution and polydisperse nature in suspension. It calculates the hydrodynamic radius of a particle undergoing Brownian motion, which is crucial for understanding its functioning. When a particle in a solution is exposed to a laser beam, it scatters light with different intensities, and the Stokes–Einstein connection equation is used to analyze these fluctuations. DLS provides reliable measurements of particle sizes ranging from 20 to 200 nm. DLS is widely used in industry and research laboratories to analyze particle size distribution profiles of nanoparticles in solutions and colloidal suspensions. Its fast and sensitive detection capabilities allow for real-time determination of nanoparticle size	141, 146 and 157

Characterizing nanoparticles is challenging due to their small size and the complexity of their interactions. No single method is ideal for all situations; the choice of method depends on the type of sample, desired information, time constraints, and economic feasibility. Therefore, a combination of methods is often used to achieve comprehensive characterization. Comparing results from different techniques can also enhance accuracy, as each method has varied sensitivity, advantages, and limitations.^145^

## 6. Heavy metals removal

**Fig. 14:**
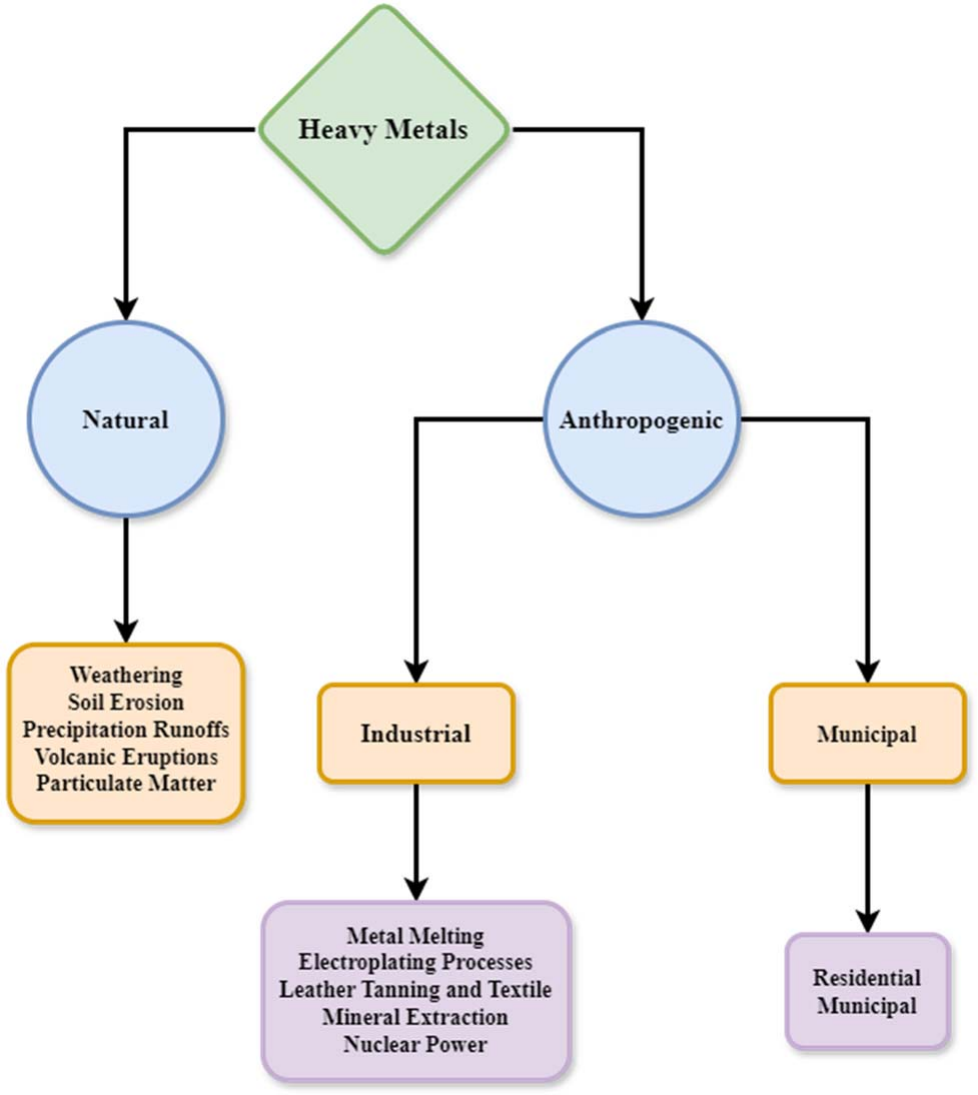
Graphical representation of heavy metal contamination sources.^162^

There is a growing trend of heavy metals being released into the environment, particularly in developing nations. Unlike organic pollutants, heavy metals are not broken down by biological processes and tend to build up in living organisms. Many heavy metal ions are recognized for their toxicity or ability to cause cancer.^160^ The atomic masses of heavy metals range from 63.5 to 200.6, and their specific gravities are more than 5.0.^161^ These elements are naturally present in the Earth's crust. Heavy metal contamination occurs due to natural phenomena and civilization. In addition to natural sources, human activities are the primary cause of heavy metal contamination. Fig. 14 provides a comprehensive summary of the primary sources of heavy metals.

**Fig. 15:**
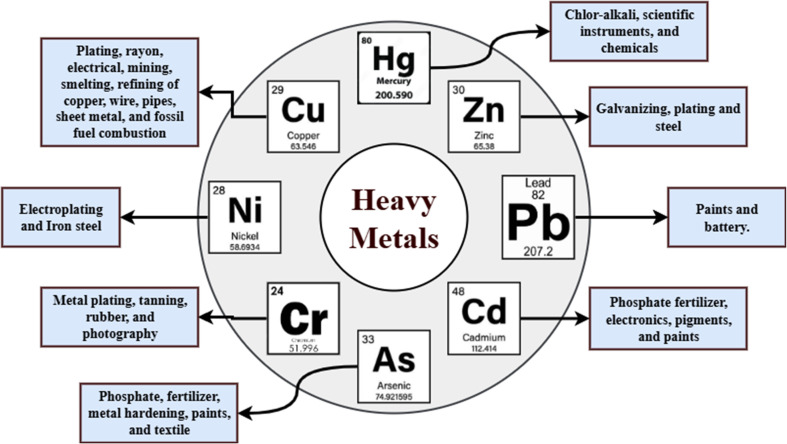
Most common heavy metals observed in industrial wastewater and their primary manufacturing origins.^174^

The extensive use of heavy metals such as cadmium, arsenic, selenium, lead, and mercury significantly impacts the hydrosphere, biosphere, lithosphere, and atmosphere.^163^ The main heavy metals detected in industrial wastewater and their common industrial manufacturing origins are presented in Fig. 15. Several heavy metals serve as essential trace elements, functioning as micronutrients for people, flora, and fauna in small quantities. However, these heavy metals may lead to short-term and long-term toxicities when present in greater quantities. Since the 1970s, authorities have increasingly focused on addressing the contamination of heavy metals and have developed laws to regulate and mitigate its impact. The European Union, formerly known as the European Community, requires the collection and remediation of municipal wastewater. Since the 1990s, it has been prohibited to dispose of this effluent directly into water bodies. The European law mandates that waste management initiatives adhere to the following priority orders: prevention, reuse, recycling, alternative forms of recovery (such as energy recovery), and disposal.^164^ The United States Environmental Protection Agency (USEPA) and the WHO have established recommendations for the maximum allowable levels of hazardous heavy metals in drinking water and industrial effluent. These regulations outline the potential adverse health effects when these regulations are exceeded. Table 6 displays the dangerous heavy metals and the maximum allowable levels for their presence in drinking water and industrial effluent, according to regulations issued by the USEPA and WHO standards. The toxic impact of heavy metals escalates with increased concentrations; hazardous heavy metals may be very harmful to humans if ingested in amounts that exceed the maximum contamination level permitted in drinking water.^165,166^ In addition to impacting people and animals, it is important to ensure that the concentration of heavy metals in plants remains below the permitted level. If this limit is surpassed, there may be severe repercussions. Hence, it is essential to remediate wastewater polluted with these hazardous metals before releasing it into the environment or reusing it in industrial processes.^93^

**Table 6:** Acceptable limits for toxic heavy metals and their adverse impact^93,160,163,165,170,175–179^

Heavy metals	Drinking water		WHO for wastewater (ppb)	Negative effects
	USEPA (ppb)	WHO (ppb)		
Chromium	100	<50	50	Diarrhea, headache, and nausea. It impacts human physiology, bioaccumulates in the food chain, and leads to significant health issues, ranging from minor irritation of the skin to the development of lung cancer
Lead	15	<10	10	Damage to the central nervous system, the kidney, the liver, the reproduction system, fundamental cell processes, and the brain's functioning
				Toxic effects include anemia, insomnia, dizziness, headaches, irritation, muscular weakness, hallucinations, and renal damage
Nickel	100	20–70	20	Conditions such as weight loss, eczema, and hair loss. Severe respiratory and renal issues, as well as liver and kidney damage, may lead to cancer in individuals; gastrointestinal discomfort, lung scarring, and skin inflammation
Copper	1300	<2000	1000	Damage to the liver and kidneys. Liver cirrhosis in individuals. Stomachache, anemia, vomiting, nausea, and headache in children
				Overconsumption of copper may lead to severe toxicological issues, including symptoms such as vomiting, cramping, convulsions, and sometimes even death
Cadmium	5	3–5	3	Disorders affecting the muscles and skeletal system; irritate the respiratory system. Prolonged exposure to cadmium leads to renal failure, and excessive exposure may be fatal
Mercury	2	<6	50	Neurological effects include tiredness, headaches, tremors, hearing and cognitive impairment, and hallucinations
				High mercury levels result in the deterioration of respiratory and renal function, which is accompanied by symptoms such as chest discomfort and difficulty breathing
Zinc	5000	<3000	2000–5000	Diarrhea, vomiting, jaundice (yellowing of the mucous membranes), hematuria (presence of blood in the urine), anemia, renal failure, and hepatic failure. Abdominal pain and dermatological discomfort
Arsenic	10	<10	N/A	Addiction may result in symptoms such as nausea and vomiting, reduced hematopoiesis, arrhythmia, and vascular damage. The individual has been diagnosed with cancer in the liver, kidneys, lungs, and bladder, as well as a skin lesion and visceral malignancies causing dermatitis

Surface water contamination with heavy metals is a widespread environmental issue worldwide. The quantities of heavy metals in Nigeria's Niger River water in 2004 were measured at 50 parts per billion (ppb) for cadmium, 30 ppb for lead, 2080 ppb for chromium, and 780 ppb for nickel. In 2013, the Korotoa River water in Bangladesh had a concentration of 11 ppb of cadmium, 35 ppb of lead, 83 ppb of chromium, and 46 ppb of arsenic. The excessive buildup, biomagnification, and toxicity of heavy metals in surface water have raised serious concerns among governments and the public.^164^ Water heavy metal concentrations above the standards set by WHO and USEPA have been observed to be lower in industrialized nations such as Europe and North America, whereas they were higher in developing countries like Asia, Africa, and South America.^164^

Several techniques are available for eliminating these heavy metals, including precipitation, filtration, adsorption, oxidation or lessening, reverse osmosis, ion exchange, and electrochemical remediation. However, these approaches lose effectiveness when heavy metals exceed 100 milligrams per liter. Many heavy metal salts exhibit water solubility and readily dissolve in wastewater, rendering them incapable of being extracted by physically separated techniques.^165,167^ Innovative methods such as nanotechnology have emerged over the past few years to address the deficiency in reusing heavy metals from wastewater for environmental purposes. Nanotechnology has been significant in the advancement of wastewater remediation and the preservation of the ecosystem. In the present situation, using nanomaterials has become essential for improving several industrial settings and processes, including catalysis, healthcare, and sensing. This eventually leads to decreased wastewater production and its handling.^168,169^ Recently, more advanced nanoadsorbents have been developed to enhance the adsorption process. On the other hand, advanced technologies like photocatalysis have shown their effectiveness and potential in eliminating heavy metals.^170,171^ Significant progress has been achieved in many nanomembrane methods, including ultrafiltration, nanofiltration, and reverse osmosis.^172,173^

## 7. Adsorption for heavy metals removal

Adsorption is a surface phenomenon characterized by the attachment of heavy metal ions or molecules to an adsorbent. The process typically occurs in two stages: (1) the initial interaction between the adsorbent and the heavy metal-contaminated aqueous solution, and (2) the subsequent separation of the heavy metal-loaded adsorbent from the treated effluent. Adsorption is conducted through two primary methods: batch and column experiments. In batch adsorption, the adsorbent is dispersed in contaminated water with continuous agitation to establish equilibrium between the solid and liquid phases. Column adsorption consists of the continuous flow of contaminated water through a packed bed of adsorbent, a method frequently utilized in industrial applications owing to its operational efficiency and scalability. The physicochemical characteristics of the adsorbent and the existing operational conditions largely determine the efficacy of the adsorption process. An effective adsorbent must possess a high specific surface area, numerous active binding sites, structural stability, and selectivity for the target metal ion, regardless of competing contaminants. Furthermore, effective adsorbents must be economically viable, non-toxic, environmentally sustainable, and able to undergo repeated regeneration and reuse with minimal performance degradation.^174^

**Fig. 16:**
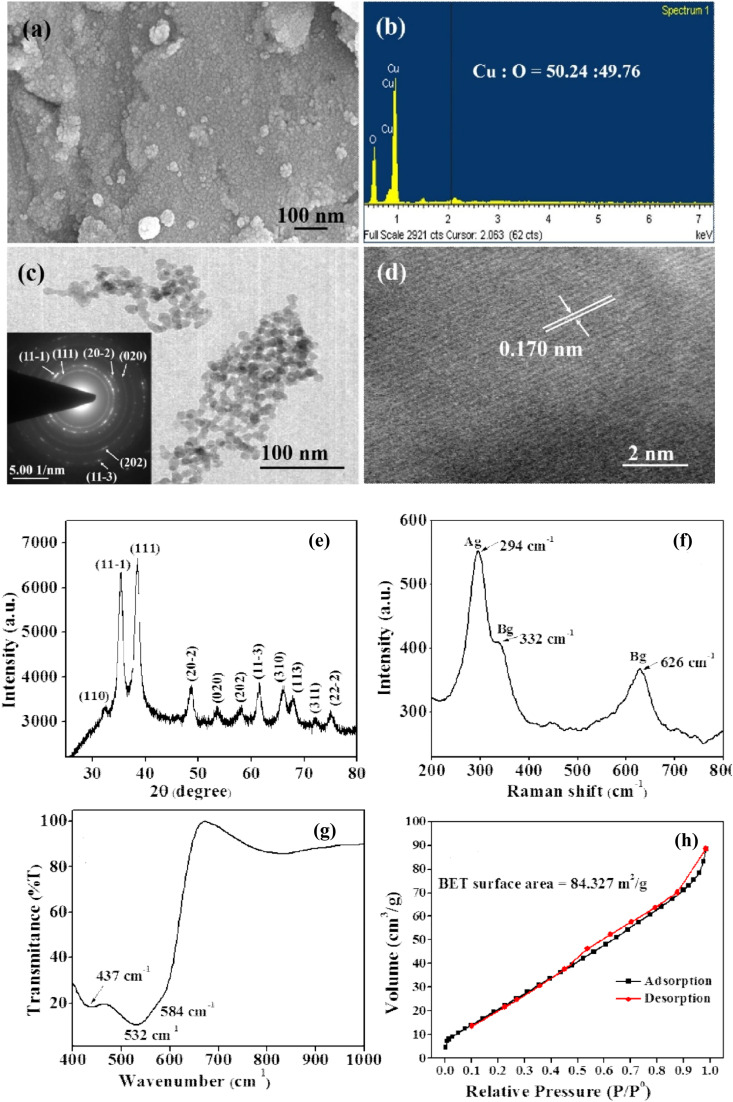
Characterization of copper oxide nanoparticles using (a) FE-SEM, (b) EDX, (c) TEM micrograph with inset displaying the SAED pattern, (d) HR-TEM, (e) XRD, (f) RS, (g) FT-IR, and (h) BET. Adapted from ref. 181 with permission from Elsevier, copyright 2016.

**Fig. 17:**
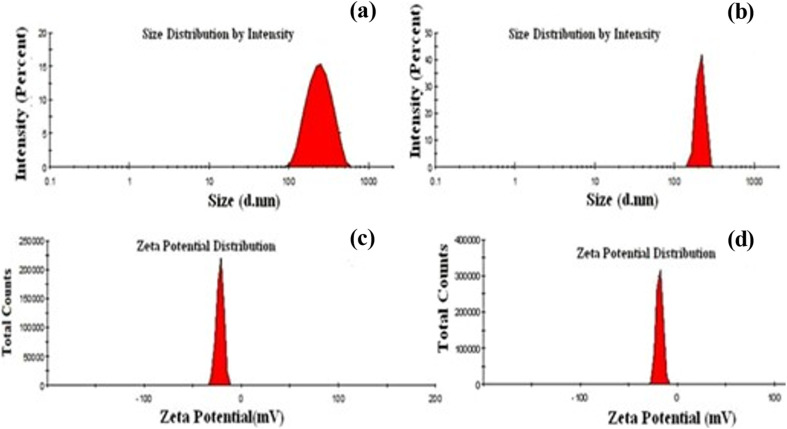
Physicochemical properties of chitosan-alginate nanoparticles: (a and b) hydrodynamic size distribution and (c and d) zeta potential before and after adsorption. Adapted from ref. 183 with permission from Elsevier, copyright 2016.

**Fig. 18:**
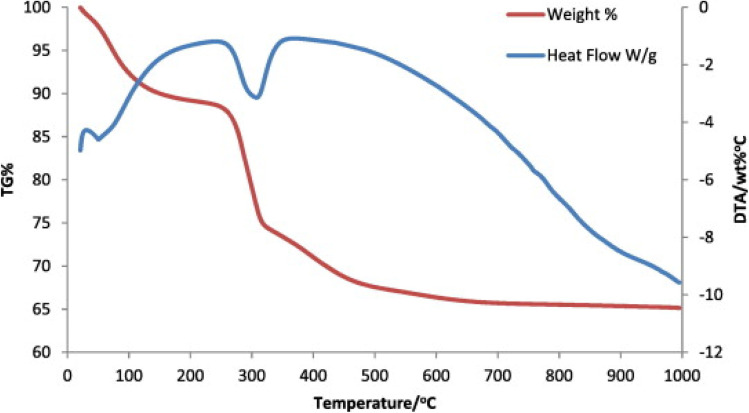
Thermal characterization of zinc sulfide nanocrystals: TGA and DTA. Adapted from ref. 185 with permission from Elsevier, copyright 2015.

**Fig. 19:**
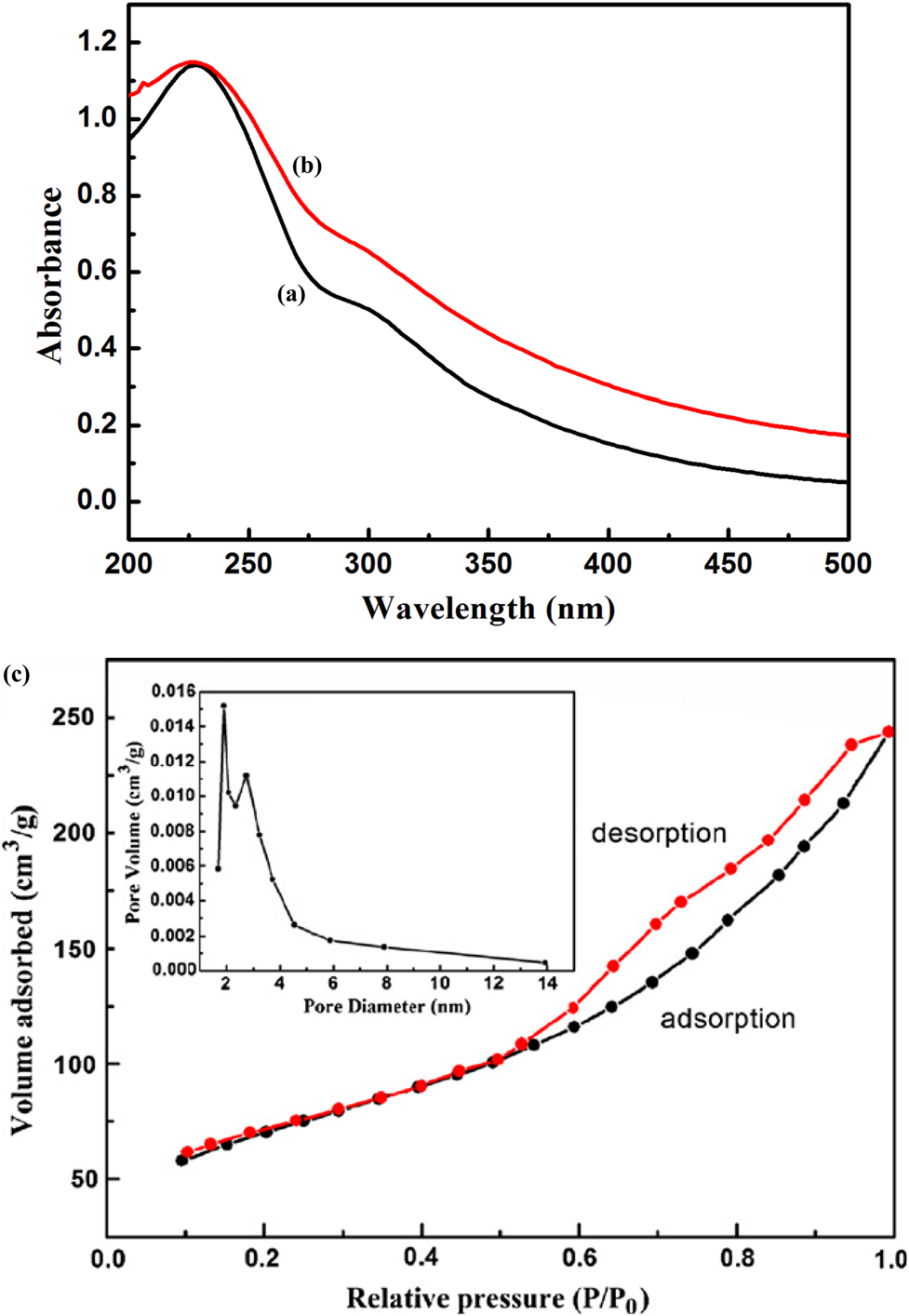
UV-Vis spectra and surface textural properties of silicon dioxide/graphene composite: using various techniques: UV-Vis of (a and b) UV-Vis absorbance of graphene oxide and silicon dioxide/graphene composite showing a blueshift upon composite formation and (c) BET analysis and pore size distribution (BJH) for silicon dioxide/graphene composite. Adapted from ref. 187 with permission from Elsevier, copyright 2012.

**Fig. 20:**
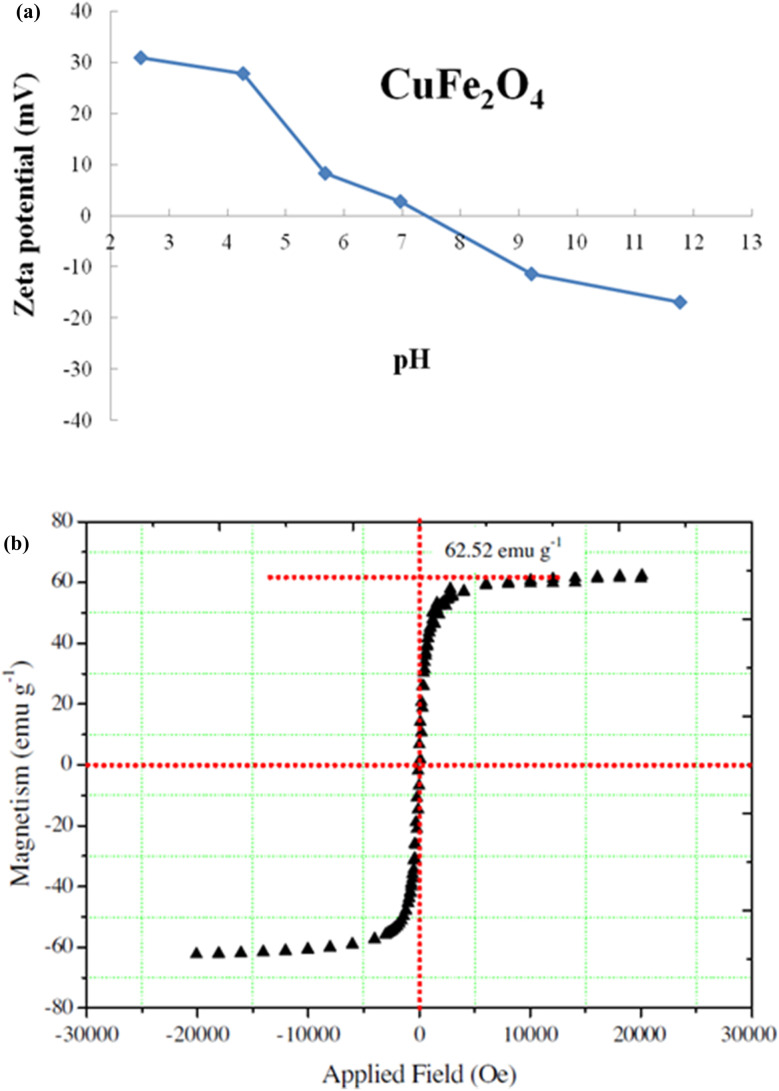
Surface and magnetic properties of copper ferrite nanoparticles: (a) pH-dependent zeta potential analysis, and (b) magnetization curves (VSM). Adapted from ref. 188 with permission from Elsevier, copyright 2017.

**Fig. 21:**
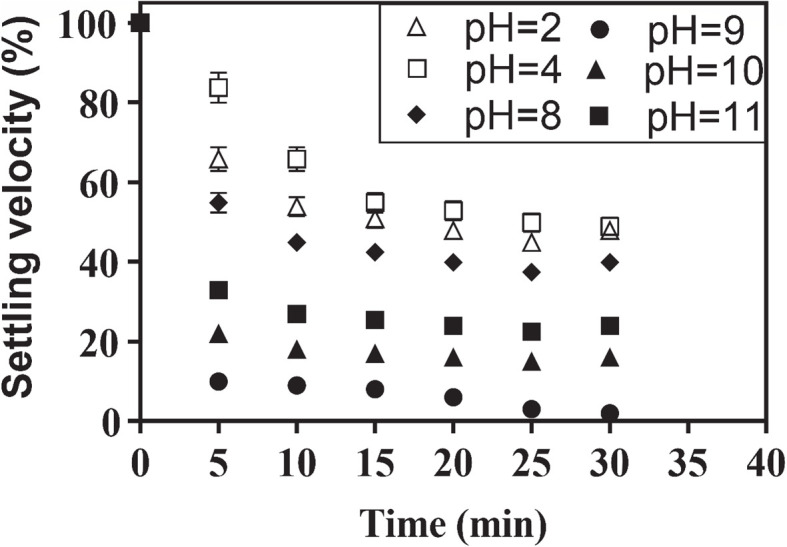
Effect on settling velocity of magnetic nanoparticles grafted on hyperbranched polyglycerol, demonstrating their pH-responsive colloidal stability and aggregation behavior in aqueous media. Adapted from ref. 209 with permission from Elsevier, copyright 2020.

Nanoadsorbents utilize nanoscale dimensions along with modified surface chemistry, resulting in a considerably higher surface-to-volume ratio, enhanced density of active binding sites, and distinct physicochemical properties that can be modified for particular applications.^180^ Researchers have investigated various nanomaterials for the adsorption of heavy metals, including carbon-based, zeolites, polymer-based, metal-based, silica, metal–organic frameworks (MOFs), and layered double hydroxides.^174,180^ The main adsorption mechanisms for these nanoadsorbents consist of surface complexation, ion exchange, electrostatic attraction, precipitation, and chelation, all of which may be affected by surface functionalization, particle morphology, and pore structure. Nanoscale engineering enables precise alterations in particle size, shape, and surface chemistry, thereby optimizing adsorption kinetics and capacity for specific heavy metals.^176,180^ Recent studies indicate that nanoadsorbents exhibit enhanced adsorption capacities, accelerated kinetics, and improved recyclability relative to traditional adsorbents, positioning them as a promising option for water and wastewater treatment. Table 7 provides a comprehensive overview of nanomaterials studied for heavy metal removal, and Fig. 16–21 display the characterization techniques utilized in recent research.

**Table 7:** Experimental parameters and removal efficiency for the elimination of heavy metal ions using different nanoadsorbents^a^

Adsorbent	Dose, milligrams per liter	Heavy metals	Initial concentration, milligrams per liter	Time, minute	pH	Temperature, °C	Characterization	Adsorption capacity		Ref.
								%	Milligrams per gram	
Magnetic nanoparticles coated mixed fungal biomass	700	Chromium(vi)	50	60	2	30	FT-IR, SEM, EDX	99.425		78 and 150
Copper(ii) oxide nanoparticles	1600	Chromium(vi)	20	10	3	25	XRD, RS, FE-SEM, EDX, TEM, HR-TEM, BET, FT-IR, AAS, SAED	96.3	15.625	180 and 181
Magnetic multiwall carbon nanotubes	1000	Chromium(vi)	2	600	3	30	TEM, XRD	100		52 and 182
Chitosan-alginate nanoparticles	200	Mercury(ii)	4	90	5	30	FT-IR, TEM, ZPA, SD, SEM, EDX		217.39	183
Zinc sulfide nanocrystals	2000	Mercury(ii)	297.5	5	1–6	22.4–34.6	TEM, XRD, EDX, HR-TEM	99.99		52 and 184
Nickel oxide nanocatalyst	1000	Lead(ii)	5	120	5.8	25	XRD, DTA, TGA, BET, FT-IR	100		175 and 185
Silicon dioxide/graphene composite	300	Lead(ii)	20	60	6	25	UV-vis, FT-IR, XPS, XRD, SEM, BET	98.82		186 and 187
Copper ferrite nanoparticles	0.02	Lead(ii)	10	120	4.5	25	BET, XRD, SEM, SQUID, ZPA		17.83	180 and 188
Carbon nanotubes	40	Lead(ii)	540	80	5	25	EDX	96.03		176 and 189
Granular ferric oxide	8000	Arsenic(iii)	30	49.99	5	25	SEM, FT-IR, pHpzc (ZPA)	66.99		175 and 190
Magnetic nanoparticles coated with zeolite	500	Arsenic(iii)	20	15	2.5	25 ± 1	TEM, EDX, FT-IR, XRD	95.6		52 and 191
Nano zero-valent iron	1000	Arsenic(iii)	1	10	7	N/A	XRD, SEM	99.9		52 and 192
Magnetic	2000	Zinc(ii)	10	90	5.5	25	SEM, EDX, XRD, FT-IR, VSM	95		52 and 156
Magnetic tubular carbon nanofibers	500	Copper(ii)	50	10	6	30	FT-IR, TGA, XRD, BET, BJH, XPS, VSM, SEM, EDX, HR-TEM, AAS, HR-EDX	99.9 ± 0.1		78 and 193
Functionalized multiwalled carbon nanotubes	10	Copper(ii)	20	60	3	25	FT-IR, XRD, SEM, TEM	93	118.41	175 and 194
Carbon nanotube/calcium alginate composites	500	Copper(ii)	5–40	120	5	20	SEM, BET, FT-IR	83.3		195
Mesoporous magnetite nanoparticles functionalized with amines	1000/3	Copper(ii)	5	30	7	25	TEM, HR-TEM, XRD, RS, BET, BJH, VSM, FT-IR, SAED	∼85		180 and 196
Sulfonated magnetic graphene oxide composite	18 ± 6.4	Copper(ii)	73.71	180	4.68	50	TEM, EDX, PS, BET, TG-DTA, FT-IR, RS		62.73	186 and 197
Modified multiwalled carbon nanotubes	1200	Nickel(ii)	50	0.6	6	N/A	RS, SEM, EDX, FT-IR, TGA	65		198
Multiwalled carbon nanotubes	500	Nickel(ii)	10–80	900	7	25	BJH, BET, FT-IR	93.4		176 and 199
Maghemite nanoparticles coated bacteria	N/A	Cadmium(ii)	10	120	4	30	AAS, SEM, EDX	84.2		78 and 200
Hybrid polymeric nanocomposite	400	Cadmium(ii)	25	1440	7	25	FT-IR, TGA, XRD, SEM, EDX, BET	72.36	45.22	52 and 201
		Zinc(ii)						66.80	41.75	
		Lead(ii)						79.6	49.72	
Magnetite nanorods	1000	Iron(ii)	50	60	5.5	25	SEM, TEM, XRD, BET	99.88	127.01	180 and 202
		Lead(ii)						99.89	112.86	
		Zinc(ii)						99.75	107.27	
		Nickel(ii)						99.65	95.42	
		Cadmium(ii)						99.75	88.39	
		Copper(ii)						99.45	79.10	
Milled goethite nanocrystalline	400	Cadmium(ii)	50	720	7 ± 0.2	25	XRD, TEM, VSM		66.11	132 and 203
		Nickel(ii)							53.11	
		Cobalt(ii)							53.91	
		Chromium(vi)							45.38	
Magnetic cobalt ferrite-reduced graphene oxide nanocomposites	160	Lead(ii)	20	80	5.3	25	XRD, BET, ZPA	87.5	299.4	186 and 204
	140	Mercury(ii)	5	60	4.6			89	157.9	
Zinc oxide nanoparticles	0.5	Zinc(ii)	100	120	5.5	30	XRD, SEM, EDX		357	41 and 205
		Cadmium(ii)							384	
		Mercury(ii)							714	
Magnetic graphene oxide	16	Lead(ii)	60	25	5	25	UV-vis, FT-IR, SEM, XRD, VSM, AAS	91.5		175 and 206
		Chromium(iii)		35	5			87.5		
		Copper(ii)		25	6			88.6		
		Zinc(ii)		35	7			87		
		Nickel(ii)		25	8			85		
Innovative nanoadsorbent composed of electrospun polyvinyl alcohol nano zeolite nanocomposite nanofibers	500	Nickel(ii)	10	60	5	45	FT-IR, SEM, BET, BJH, ICP-AES	90	838.7	180 and 207
		Cadmium(ii)						82	342.8	
Maghemite nanoparticles functionalized with homopolymers of a novel mercaptoethylamino monomer	1500	Cadmium(ii)	20	100	6	25	FT-IR, XRD, BET, TEM, pHpzc		91.5	180 and 208
		Mercury(ii)							237.6	
		Lead(ii)							118.5	
		Silver(i)							260.5	
Magnetic nanoparticles – hyperbranched polyglycerol	30	Nickel, aluminum, and copper	48	130	9	20	SV, TEM, DLS, XPS, XRD, FT-IR, VSM, ICP-AES	94		78 and 209
Carbonate-based mesoporous magnetic	2000	Arsenic(v)	10	9	2	25	UV-vis, ICP-MS, UHR-FESEM, BET, BJH, XRD, FT-IR, XPS, FE-SEM, STEM, EDX	99.99	184.1	180 and 210
		Chromium(vi)							251.6	
		Lead(ii)							1041.9	
2,4-Dinitrophenylhydrazine-modified nano-alumina	1200	Lead(ii)	50	90	5	25 ± 1	FT-IR, SEM, EDX, BET, XRD, AAS	97.25		41 and 211
		Cadmium(ii)						74.17		
		Nickel(ii)						36.26		
		Cobalt(ii)						53.29		
		Manganese(ii)						10.98		
		Chromium(iii)						91.76		

a AAS = Atomic Absorption Spectrometry; BET = Brunauer–Emmett–Teller; BJH = Barrett–Joyner–Halenda; DLS = Dynamic Light Scattering; DTA = Differential Thermal Analysis; EDX = Energy Dispersive X-ray; FE-SEM = Field Emission Scanning Electron Microscopy; FT-IR = Fourier Transform Infrared Spectroscopy; HR-EDX = High-Resolution Energy Dispersive X-ray; HR-TEM = High-Resolution Transmission Electron Microscopy; ICP-AES = Inductively Coupled Plasma Atomic Emission Spectroscopy; ICP-MS = Inductively Coupled Plasma Mass Spectrometry; pHpzc = point of zero charge; PS = Particles Size; RS = Raman Spectroscopy; SAED = Selected Area Electron Diffraction; SD = Size Distribution; SEM = Scanning Electron Microscope; STEM = Scanning Transmission Electron Microscopy; SV = Settling Velocity; SQUID = Superconducting Quantum Interference Device; TEM = Transmission Electron Microscopy; TGA = Thermogravimetric Analysis; TG-DTA = thermogravimetric-differential thermal analysis; UHR-FESEM = Ultra-High Resolution Field Effect Scanning Electron Microscope; UV-vis = ultraviolet-visible spectrophotometer; VSM = Vibrating-Sample Magnetometry; XPS = X-ray Photoelectron Spectroscopy; XRD = X-Ray Diffraction; ZPA = Zeta Potential Analyzer.

A comparative analysis of the nanoadsorbents listed in Table 7 reveals significant variability in adsorption performance. For instance, innovative electrospun polyvinyl alcohol nano zeolite nanocomposite nanofibers achieved an exceptionally high adsorption capacity of 838.7 mg g^−1^ for nickel(ii) at 45 °C and Ph 5, outperforming traditional materials like carbon nanotubes, which showed a 96.03% removal efficiency. Similarly, magnetite nanorods demonstrated consistent high uptake across multiple metal ions, suggesting their versatility and robustness. However, materials like granular ferric oxide required much higher dosages (8000 mg L^−1^) for moderate removal efficiency (66.99%), indicating lower cost-effectiveness. The structure–activity relationship is evident when comparing materials with high BET surface areas and functionalized surfaces. For example, copper oxide nanoparticles (Fig. 16) exhibited a BET surface are of 84.327 m^2^ g^−1^, contributing to their high adsorption capacity of 15.625 mg g^−1^ for chromium(vi). Similarly, sulfonated magnetic graphene oxide composites exhibited enhanced adsorption due to the presence of sulfonic functional groups, despite a lower dosage. Zeta potential analysis (Fig. 20) further supports the role of surface charge in metal ion affinity, with materials exhibiting more negative zeta potentials at optimal pH showing better adsorption performance. While high-performance nanoadsorbents like maghemite nanoparticles functionalized with homopolymers show promising uptake values (*e.g.*, 260.5 mg g^−1^ for silver(i)), their synthesis complexity and potential environmental risks (*e.g.*, nanoparticle leaching) pose scalability challenges. Materials requiring extreme pH conditions (*e.g.*, pH 2 for arsenic removal) may not be viable for large-scale systems. Moreover, regeneration efficiency varies widely; some polymer-based adsorbents retain performance over multiple cycles, while others degrade rapidly. Engineering integration remains a bottleneck, especially for materials requiring long contact times or high dosages.

### 7.1 Carbon-based nanoadsorbents

Carbon-based nanomaterials have been shown to be very efficient adsorbents for the removal of heavy metals from polluted water, owing to their extremely high specific surface area, adjustable surface chemistry, mechanical strength, and strong adsorption affinity.^212,213^ Their nanoscale size provides a high density of active sites that may interact with heavy metal ions, including Pb^2+^, Cr^6+^, Cd^2+^, Cu^2+^, Hg^2+^, and Ni^2+^, *via* electrostatic attraction, ion exchange, surface complexation, and π–π interactions.^212,214^ In comparison to traditional adsorbents, carbon nanostructures provide enhanced performance due to their distinctive shape, elevated reactivity, and simple functionalization.^213^

Carbon nanotubes (CNTs), graphene-based materials, fullerenes, and activated carbon are extensively researched. CNTs may be categorized as single-walled CNTs or multi-walled CNTs cylindrical hollow structures composed of rolled graphene sheets, may demonstrate theoretical specific surface areas of 2600 m^2^ g^−1^; however, aggregation in aqueous environments can reduce this value.^174,214^ Functionalization with oxygen-containing (–OH, –COOH, –CO) or nitrogen (–NH_2_) groups enhances dispersibility, stability, and metal-binding affinity. For instance, functionalized multi-walled CNTs demonstrated over 90% removal efficiency for Pb^2+^ and Cd^2+^, while oxygenated single-walled CNTs reached over 95% removal of Hg^2+^ and As^3+^. The reported adsorption capabilities for multi-walled CNTs are 88.62 mg g^−1^ for Cd^2+^ and 90.90 mg g^−1^ for Zn^2+^ under optimum situations.^212,214^ Graphene and its derivatives, especially graphene oxide, received considerable attention owing to their two-dimensional architecture, extensive surface area, and many oxygenated functional groups that enhance dispersion and adsorption in aqueous solutions.^180,215^ Graphene oxide interacts with heavy metal ions mostly *via* complexation at oxide binding sites, exhibiting capacities of 243.9 mg g^−1^ for Zn^2+^ (ref. 214) and 406.6 mg g^−1^ for Pb^2+^.^215^ Reduced graphene oxide provides significant adsorption capabilities while providing enhanced conductivity and stability. Fullerenes (*e.g.*, C_60_, C_70_) are spherical carbon allotropes characterized by strong electron affinity, hydrophobic surfaces, and substantial surface-to-volume ratios, facilitating the adsorption of organic and inorganic pollutants. Research indicates the efficient adsorption of Cu^2+^ and other heavy metals, often according to the Langmuir isotherm, with improved efficacy when integrated into composites with activated carbon, lignin, or zeolites.^215,216^ Activated carbon is among the most prevalent carbon-based adsorbents, attributed to its highly developed porous architecture, extensive surface area, and comparatively inexpensive production cost from biomass. Activated carbon produced from eucalyptus bark, chicken litter, or rubberwood sawdust has adsorption capabilities that surpass those of most commercial coal-based activated carbons. Eucalyptus bark-derived activated carbon attained 0.45 mmol g^−1^ for Cu^2+^ and 0.53 mmol g^−1^ for Pb^2+^, while rubberwood sawdust-derived activated carbon obtained 44 mg g^−1^ for Cr^6+^ at pH 2.^215^

In addition to pure carbon nanomaterials, carbon-based nanocomposites have developed as a promising category of adsorbents that combine carbon structures with functional nanoparticles, polymers, or metal oxides to improve adsorption capacity, selectivity, and regeneration potential. This encompasses composites of activated carbon, CNTs, graphene, or biochar combined with metal nanoparticles like zero-valent iron, silver, titanium dioxide, or manganese oxides. The combination enhances performance by offering additional reactive sites, promoting redox transformations, and increasing selectivity *via* functional groups such as –COOH, –OH, and –SO_3_H. Zero-valent iron-carbon composites can transform Cr(vi) to the less hazardous Cr(iii), which subsequently precipitates from the solution.^212^ Numerous studies have proven the superior performance of these composites. Polyethyleneimine-functionalized multi-walled CNTs obtained 99% Cr(vi) removal (adsorption capacity of 40 mg g^−1^) after 60 minutes at pH < 4 and maintained 80% efficiency after five reuse cycles. Polydopamine and polyethyleneimine-functionalized CNTs showed a copper ion (Cu^2+^) adsorption capacity of 70.9 mg g^−1^, while poly(amidoamine) dendrimer-modified CNTs attained remarkable capacities of 4870 mg g^−1^ for lead ions (Pb^2+^) and 3333 mg g^−1^ for copper ions (Cu^2+^) *via* metal-amine coordination.^214^ CNT-based hybrids including TiO_2_ and MnO_2_ have capacities of 137 mg g^−1^ and 78.74 mg g^−1^ for Pb^2+^, respectively.^215^ Graphene oxide composites have improved performance; for instance, montmorillonite/graphene composites achieved 247.85 mg g^−1^ for Pb^2+^.^180^ Biochar-based composites, typically produced from biomass pyrolysis, provide a sustainable and economical option. The integration of iron or manganese oxides into biochar enhances heavy metal adsorption and promotes the reduction of hazardous metal species, making them suitable for extensive purposes. Chitosan–carbon nanocomposites have shown efficacy, attaining 54.6% heavy metal removal while being cost-effective and scalable.^212^

Carbon-based adsorbents and their nanocomposites provide a diverse and high-performance alternative for heavy metal remediation in water. Although CNTs and graphene have exceptional adsorption capabilities, composites that include functional nanoparticles or polymers may further improve efficiency, selectivity, and regeneration potential. Outstanding issues include the prevention of nanoparticle leaching, the enhancement of large-scale recovery, and the reduction of manufacturing expenses. Continuous progress in economical synthesis, environmentally friendly functionalization, and hybrid material design is anticipated to enhance their utilization in sustainable water treatment systems.^180,212–216^

### 7.2 Zeolite

Zeolites are crystalline microporous aluminosilicate characterized by a three-dimensional framework of SiO_4_ and AlO_4_ tetrahedra linked by shared oxygen atoms.^217,218^ The isomorphic substitution of Si^4+^ by Al^4+^ generates a net negative charge on the framework, which is balanced by exchangeable cations (*e.g.*, Na^+^, K^+^, Ca^2+^) located within the well-defined channels and cages. This unique structure underpins their high cation exchange capacity (CEC), molecular sieving ability, and reversible dehydration, making them highly effective adsorbents.^62,215,217^ Naturally occurring zeolites such as clinoptilolite and chabazite are widely used in water treatment due to their abundance, low cost, ecological sustainability, and inherent selectivity for heavy metals like Pb^2+^, Cd^2+^, and Cu^2+^.^215–217^

The primary mechanism for heavy metal removal is ion exchange, in which aqueous metal ions replace charge-balancing cations in the zeolite framework. This process is complemented by surface complexation and physical adsorption within the porous network.^213,215,219^ The adsorption capacity is strongly influenced to the Si/Al ratio, which determines hydrophobicity, acidity, and stability. Low-silica zeolites (Si/Al < 2) are hydrophilic, while high-silica zeolites (Si/Al > 5) exhibit greater thermal stability and resistance to chemical attack.^217^ However, structural stability remains a limitation; the aluminosilicate framework is susceptible to dealumination under acidic conditions, leading to crystalline collapse, loss of porosity, and permanent reduction in adsorption capacity.^219^ This acid sensitivity can limit their use in treating acidic waste streams without pre-neutralization. Furthermore, the presence of mineral impurities in natural zeolites can reduce their effective surface area and cation exchange capacity, while their performance is also highly dependent on solution pH, which affects both surface charge and metal speciation.^219,220^

To overcome these limitations and enhance performance, several modification strategies have been developed. Chemical treatments with acidic, bases, or salt solutions (*e.g.*, NaCl) can remove impurities, alter the Si/Al ratio, and enhance ion-exchange capacity.^219^ Nano-structuring zeolites increases the surface-area-to-volume ratio, reduces diffusion path lengths, and improves adsorption kinetics and capacity for heavy metals.^62,180,215^ NaX nanosized zeolite has been extensively used for the removal of Cd^2+^, exhibiting enhanced efficacy relative to bulk zeolite.^215^ Functionalization with nanoparticles or specific groups significantly improve adsorption performance.^212,213^ Magnetic zeolite nanocomposites (*e.g.*, Fe-zeolite) integrate the substantial adsorption capacity of zeolites with the magnetic separation properties of iron oxide, facilitating the effective elimination of Pb^2+^, Cd^2+^, and As^3+^. Manganese-zeolite composites enhance redox characteristics, enabling the reduction of poisonous Cr^6+^ to less dangerous Cr^3+^, subsequently followed by adsorption. Copper–zeolite composites enhance surface reactivity and form stable complexes with Hg^2+^, and Pb^2+^.^212^ Ag-modified zeolites release Ag^+^ ions, providing antibacterial characteristics while maintaining heavy metal adsorption capability.^62^

Regeneration is essential for sustainable application of zeolites. Chemical regeneration with dilute acids or salts may replenish ion-exchange sites while maintaining structural integrity, enabling repeated application over several adsorption–desorption cycles. However, acid regeneration must be carefully controlled to avoid structural degradation through dealumination.^212,219^ When properly regenerated, zeolites retain high efficiencies across multiple cycles, making them suitable for continuous industrial applications. Under optimized conditions, natural zeolites have achieved up to 99% removal of Cu^+2^ at neutral pH, underscoring their practical potential for cost-effective and sustainable water purification.^212,216^

### 7.3 Metal-based nanoparticles

Metal-based nanoparticles have shown to be very efficient materials for eliminating of heavy metals and other pollutants from water and wastewater owing to their elevated surface-to-volume ratio, increased reactivity, and adjustable surface chemistry.^212^ Their small particle size provides many active sites for adsorption, ion exchange, and catalytic processes, while their physicochemical characteristics may be modified using synthesis methods such as chemical reduction, green synthesis, and surface functionalization.^212,213^ Iron-based and silver nanoparticles are the most thoroughly researched and used in the treatment of water and wastewater for the removal of heavy metals. The adsorption mechanisms of metal-based nanoparticles are primarily governed by the physicochemical properties of the metal oxide, the nature of surface functional groups, and the specific characteristics of the target ions. Typical removal pathways include adsorption, ion exchange, surface complexation with hydroxyl or oxygenated groups, co-precipitation, redox reactions, and electrostatic attraction. In semiconductor oxides such as TiO_2_, ZnO, and CeO_2_, photocatalytic reduction and oxidation can also contribute significantly to metal ion immobilization. For instance, MgO, Al_2_O_3_, and TiO_2_ nanoparticles exhibit adsorption capacities of approximately 594.9, 114.6, and 49.4 mg g^−1^ for Cd^2+^, Cu^2+^, Ni^2+^, and Pb^2+^ in multicomponent systems, with MgO acting mainly through precipitation and adsorption, whereas TiO_2_ and Al_2_O_3_ primarily operate *via* surface adsorption and complexation mechanisms.^221^

Nanoscale zero-valent iron (nZVI) has been extensively studied for its efficacy in eliminating harmful heavy metals, include Cr(vi), Cd(ii), Cu(ii), Hg(ii), Ni(ii), and Pb(ii), from polluted water. Their removal methods including adsorption, electrostatic attraction, redox transformations, co-precipitation, and surface complexation.^212^ nZVI generally displays a core–shell structure, including a zero-valent iron core surrounded by an oxide shell that contains Fe(ii) and Fe(iii) species. The metallic core functions as an effective reducing agent, capable of converting more hazardous species (*e.g.*, Cr(vi)) into less dangerous forms (*e.g.*, Cr(iii)), while the oxide shell offers reactive sites for adsorption.^213^ Research has shown the remarkable efficacy of iron-based nanoadsorbents in both batch and continuous systems, as well as *in situ* groundwater remediation. Biochar-supported nZVI has improved adsorption capability and dispersion, while green-synthesized iron nanoparticles derived from tea extract have effectively treated acid mine drainage *via* adsorption, reduction, and co-precipitation.^212^ Moreover, iron oxide nanoparticles, including magnetite (Fe_3_O_4_), maghemite (γ-Fe_3_O_4_), hematite (α-Fe_3_O_4_), goethite (α-FeOOH), and amorphous hydrous iron oxides, have been widely used for the removal of heavy metals owing to their cost-effectiveness, magnetic recoverability, and environmental compatibility.^214,216^ Surface modification is crucial for improving the dispersion, stability, and selectivity of iron nanoparticles. Functional groups such as –NH_2_ and –COOH may enhance their chelation capacity and adsorption efficacy. For instance, –NH_2_-functionalized Fe_3_O_4_ nanoparticles eliminated 97.94% of Cr(vi) and 98.56% of Ni(ii) from 1 ppm tap water, exhibiting maximal adsorption capacities of 222.12 and 232.15 mg g^−1^, respectively. Also, Fe_3_O_4_@SiO_2_–NH_2_ composites attained maximum removal of Zn(ii) from industrial pickling waste, exhibiting an adsorption capacity of 169.5 mg g^−1^.^214^ In a separate investigation, Fe_3_O_4_@SiO_2_@carboxyl-PAMAM nanocomposites exhibited adsorption capacities of 117.0, 119.16, and 115.82 mg g^−1^ for Pb(ii), Cu(ii), and Cd(ii), respectively, achieving removal efficiencies above 96%.^222^ The magnetic properties of these materials enable straightforward separation and reutilization, enhancing their economic and environmental sustainability.^62^

Silver nanoparticles (Ag NPs) represent a significant category of metal-based nanoparticles, offering dual advantages in water purification: adsorption of heavy metals and antibacterial properties.^212,213^ Ag NPs have significant bactericidal characteristics *via* the production of Ag^+^ ions, which interfere with cellular activities in microorganisms, making them effective for the simultaneous elimination of infections and chemical pollutants.^212^ Their small dimensions and elevated surface area promote effective binding of hazardous metal ions, including Hg(ii), Cu(ii), Cd(ii), and Pb(ii).^213,216^ Multiple studies have shown the efficacy of Ag NPs in eliminating harmful metals from water and wastewater. Silver nanoparticles developed from microalgae and cyanobacteria extracts have shown efficient removal of iron and manganese ions.^212^ In a research study, Ag NPs exhibited a maximum Cu(ii) adsorption capacity of 25.25 mg g^−1^, while another investigation revealed a Cd(ii) capacity of 19.6 mg g^−1^. Nanocomposites based on Ag NPs have been produced to improve performance; for instance, Ag NPs combined with quartz using a sol–gel process eliminated almost 96% of Hg(ii) after 60 minutes at pH 6, conforming to the Langmuir isotherm and pseudo-second-order kinetics.^216^ In addition to adsorption, Ag NPs have been integrated into multifunctional membranes for simultaneous pollutant elimination and antibacterial efficacy. A bio-nanomembrane consisting of graphene oxide, natural rosin, Ag NPs, and a chitosan/PVA matrix demonstrated enhanced tensile strength, antibacterial characteristics, and heavy metal adsorption capabilities.^212^ Although Ag NPs are often considered low in toxicity relative to several other metals, concerns over their possible environmental discharge and accumulation persist, hence requiring regulated application and recovery techniques.^62^

In conclusion, metal-based nanoparticles, specifically iron and silver nanostructures, exhibit elevated adsorption capabilities, adjustable reactivity, and multifunctionality for eliminating of heavy metals and related contaminants from water and wastewater. Iron-based nanoparticles demonstrate superiority in redox-mediated detoxification and magnetic recoverability, whereas Ag NPs provide combination antibacterial and adsorption properties. Ongoing progress in surface functionalization, composite development, and eco-friendly synthesis is anticipated to enhance their efficacy, durability, and sustainability in practical water treatment applications.^212–214,216,222^

### 7.4 Silica nanomaterials

Silica nanoparticles (SiO_2_) are esteemed for their non-toxic nature, extensive surface area, exceptional chemical and thermal stability, mechanical strength, and easy surface functionalization, rendering them viable options for eliminating of heavy metals from aqueous environments.^212,214,216,223^ Their three-dimensional porous structure promotes fast mass transfer, while the plentiful surface silanol (Si–OH) groups serve as active adsorption sites, facilitating chelation and complexation with metal ions.^214,216^ The qualities have been further used in the formation of silica/metal nanocomposites, which integrate the adsorption, ion-exchange, and redox characteristics of metal nanoparticles with the structural stability and dispersibility afforded by the silica matrix.^212^ Silica/metal nanocomposites have significant efficacy in the removal of diverse hazardous metal ions. Iron–silica nanocomposites successfully eliminate arsenic (As^3+^) and lead (Pb^2+^), with irone component offering reactive adsorption and reduction sites, while the silica framework enhances nanoparticle dispersion and inhibits agglomeration. Manganese–silica nanocomposites may similarly decrease hazardous chromium (Cr^6+^) to less detrimental Cr^3+^ state, subsequently followed by adsorption. Copper–silica nanocomposites have a strong affinity for mercury (Hg^2+^) and cadmium (Cd^2+^) by active adsorption and surface complexation.^212^ The addition of silica improves the reactivity and durability of these composites while preventing metal nanoparticles against leaching, particularly in low pH environments.^216^

The dominant mechanisms governing metal uptake typically include precipitation, electrostatic attraction, cation exchange, and surface complexation. Precipitation occurs through the formation of insoluble hydroxides or silicates on or within the silica surface, while electrostatic attraction depends on pH relative to the point of zero charge (pHpzc), where negatively charged silica surfaces favor cation adsorption. Cation exchange involves the replacement of surface-bound alkali or alkaline earth metals (*e.g.*, Na^+^, Ca^2+^) with target metal ions, and surface complexation results from the coordination of metal ions with oxygenated functional groups such as hydroxyl or carboxyl groups. Among these, precipitation and electrostatic attraction are often predominant in fabricated nano SiO_2_ systems, while surface complexation becomes significant in functionalized or hybrid silica materials.^224^ Surface functionalization of silica nanoparticles provides an efficient approach to enhance the adsorption capacity for heavy metals. Amin (–NH_2_), thiol (–SH), phosphonate, carboxyl (–COOH), poly(ethylene glycol), and long-chain alkyl groups have been grafted onto silica surfaces to provide particular binding sites and decrease aggregation in aqueous solutions.^214^ Ethylenediaminetetraacetic acid (EDTA)-functionalized silica adsorbents had a remarkable Pb^2+^ removal capability of 1.51 mol Pb^2+^ per mol adsorbent, exceeding previous functionalized variations due to their elevated metal ion affinity and geometric flexibility.^216^ These functional modifications substantially improve adsorption capacity by enhancing the quantity and strength of binding sites.

Experimental investigations have validated the superior efficacy of silica nanoparticles in the removal of heavy metals. Bar silica nanoparticles attained a 99.5% removal of Zn^2+^ from electrically enhanced membrane bioreactor effluents in 4 hours at pH 8.2, exhibiting an adsorption capability of 9.1 mg g^−1^ as per the Langmuir model. In another instance, mesoporous silica–calcium phosphate nanocomposites, synthesized by a one-spot technique, exhibited a surface area of 314.56 m^2^ g^−1^ and a particle size of around 20 nm, successfully eliminating 99.65% of Cd^2+^ within 30 minutes at pH 6, reaching an adsorption capacity of 125.63 mg g^−1^. The composite maintained around 80% of its removal effectiveness after three adsorption–desorption cycles, demonstrating excellent reusability.^214^ Notwithstanding their superior adsorption characteristics, silica nanoparticles have an affinity to agglomerate in aqueous environments, therefore reducing their effective surface area and limiting large-scale utilization.^214^ This problem may be minimized by integrating silica into nanocomposite structures or implementing surface modifications to enhance dispersion stability. Silica nanoparticles and their composites provide a diverse and highly adaptable platform for the efficient elimination of hazardous heavy metals from water. Further study into functionalization techniques, composite fabrication, and regeneration processes is expected to improve their efficacy and facilitate their implementation in sustainable wastewater treatment systems.^212,214,216^

### 7.5 Polymer-based nanoadsorbents

Polymer-based nanoadsorbents have developed as a diverse and efficient category of materials for the elimination of heavy metals and other pollutants from water and wastewater. These nanocomposites combine the benefits of a polymer matrix, such as mechanical stability, flexibility, and adjustable surface chemistry, with the increased reactivity and extensive surface area of nanoparticles, resulting in synergistic adsorption performance that individual components cannot achieve.^212^ Commonly utilized nanoparticles encompass titanium dioxide (TiO_2_), zinc oxide (ZnO), silver (Ag), magnetite (Fe_3_O_4_), and various metal or metal oxide nanomaterials, which provides numerous active sites for adsorption, catalytic activity, and in certain instances, photocatalytic degradation, while the polymer matrix ensures structural integrity and promotes the dispersion of nanoparticles.^212,216^

Synthetic and natural polymers have been extensively investigated in polymer-based nanocomposites. Synthetic polymers, including polystyrene (PS), polyaniline (PANI), polyethylene, and polyvinyl alcohol (PVA), are widely recognized for their chemical resilience, processability, and adjustable functionalization capabilities.^216^ Natural polymers, especially biopolymers like chitosan, cellulose, and alginate, have become popular due to their biocompatibility, biodegradability, and the presence of many functional groups that may bind metal ions. Chitosan, obtained from the deacetylation of chitin (a byproduct of shellfish processing), has amino groups capable of chelating metal ions *via* coordination, ion exchange, end electrostatic attraction. Chitosan's pH sensitivity and restricted mechanical strength are often mitigated by chemical modification or the integration of nanoparticles. Chitosan/magnetite nanocomposites efficiently eliminate Pb^2+^, Cd^2+^, and Cr(vi), offering the benefit of magnetic recovery, while chitosan/silver nanocomposites integrate heavy metal removal with antibacterial properties.^180,212^ Cellulose-based nanocomposites, using renewable resources and hydroxyl functionalities, have been modified with iron oxide or silver nanoparticles to improve mechanical characteristics and adsorption efficacy for As^3+^, Hg^2+^, Pb^2+^, and microbiological pollutants.^212,216^

Polymer-based nanocomposites including dendrimer-based adsorbents, characterized by highly branched, monodisperse macromolecules including many terminal functional groups. These dendrimers can chelate heavy metals *via* the outer branches and absorb organic contaminants *via* hydrophobic cavities. Research demonstrated that dendrimer-assisted ultrafiltration effectively eliminated Cu^2+^ ions from aqueous solution, underscoring its potential in integrated treatment techniques. Despite their extraordinary efficacy, dendrimers undergo obstacles in large-scale implementation owing to their complicated and expensive manufacturing.^62^ Additional polymer nanocomposite systems are clay/polymer/metal hybrids, whereby layered clays like montmorillonite or kaolinite are intercalated with nanoparticles (*e.g.*, Fe, Zn, TiO_2_). These composites use clay's high cation exchange capacity and surface area, in combination with nanoparticles reactivity, to eliminate heavy metals *via* adsorption, ion exchange, reduction, and complexation. Iron–clay nanocomposites have been reported to decrease Cr(vi) to Cr(iii) while adsorbing Pb^2+^ and As^3+^, while Zn–clay composites have a strong affinity for Cd^2+^ and Pb^2+^.^212^

Polymer-based nanoadsorbents function *via* many processes, such as surface complexation, ion exchange, electrostatic attraction, chelation, and, in some instances, photocatalytic degradation when photoactive nanoparticles are included.^180,212^ Their adsorption performance is affected by variables like pH, contact duration, nanoparticle loading, polymer modification, and the existence of competing ions. These materials have shown superior performance in both batch and continuous flow systems, often reaching high removal efficiencies for Pb^2+^, Hg^2+^, Cd^2+^, and As^3+^.^212,216^ Furthermore, their regenerative capability *via* simple desorption techniques increases their cost-effectiveness and sustainability. Nano chitosan synthesized by polymerization with malonic acid had a maximal Pb^2+^ adsorption capacity of 32.2 mg g^−1^ at pH 6, conforming to the Langmuir isotherm and pseudo-second-order kinetics, while maintaining efficiency throughout several cycles.^216^ Polymer-based nanoadsorbents provide a viable platform for enhanced water remediation, integrating the functionality of customized polymer matrices with the high reactivity of nanomaterials. Current research aims to develop “smart” polymer nanocomposites that may react to environmental stimuli, increase selectivity for target pollutants, and improve reusability, thereby facilitating their use in sustainable heavy metal removal methods.^180,212,216^

### 7.6 MOF nanocomposites

Metal–organic frameworks (MOFs) are an innovative class of porous crystalline materials constructed from metal ions or clusters coordinated with organic ligands. They form extensive two- or three-dimensional networks characterized by exceptionally high surface area (typically 1000–10 000 m^2^ g^−1^), high porosity, tunable pore architectures.^174,225,226^ This versatility allows precise manipulation of pore geometry, surface functionality, and chemical environments, enabling selective adsorption of heavy metal ions through mechanisms such as coordination bonding, electrostatic interactions, surface complexation, and π–π interactions.^174,212^ MOFs can be classified as cationic, neutral, anionic, and bioMOFs, with charged frameworks incorporating counter-ions within pore channels to maintain electroneutrality. A wide range of metals, including Cu(ii), Al(iii), Mg(ii), Ca(ii), Fe(iii), Cd(ii), Co(ii), Zn(ii), Zr(iv), Ti(iii), and lanthanides, have been employed as framework nodes, coordinated with organic linkers such as carboxylates, phosphonates, sulfonates, or amines. To date, over 20 000 MOF structures have been synthesized and investigated for applications in gas storage, catalysis, and environmental remediation.^174^

While pristine MOFs demonstrate remarkable heavy metal removal capacity, their performance can be further enhanced by developing MOF-based nanocomposites with functional nanoparticles or polymers. For example, ZnO/MOF composites synergistically integrate the increased porosity and surface area of MOFs with photocatalytic and antibacterial properties of ZnO nanoparticles, facilitating simultaneous adsorption and elimination of metal-laden contaminants. ZnO/MOF nanocomposites exhibit rapid and effective adsorption of Cd^2+^, Pb^2+^, and Hg^2+^ ions, exceeding the performance of the individual constituents. Similarly, CuO/MOF composites utilize the reactive characteristics of CuO nanoparticles to enhance heavy metal binding and facilitate catalytic removal.^212^ Magnetic MOF nanocomposites have attracted particular attention for their efficient removal easy recovery of heavy metal-laden adsorbents. For instance, a polyacrylic acid-capped Fe_3_O_4_–Cu–MOF hybrid demonstrated outstanding selectivity for Pb^2+^, with an adsorption capacity of 610 mg g^−1^ and 93% removal efficiency in aqueous systems.^174^ The incorporation of metal nanoparticles into MOF not only enhances structural stability and prevents aggregation, but also introduces additional active binding sites, collectively improving adsorption efficacy.^212^ MOFs and MOF-derived nanocomposites have been successfully employed to remove hazardous metals including As(iii), As(v), Cr(vi), Cd(ii), Ni(ii), Pb(ii), and Hg(ii).^174,212^ Compared with conventional adsorbents such as activated carbon, zeolites, and metal oxides, they offer superior adsorption capacities, higher tunability, and better selectivity under competitive multi-ion conditions. Several pilot-scale studies highlight their potential: ethylenediamine-functionalized Zr-based MOFs achieved adsorption capacities up to 243.9 mg g^−1^ for Pb^2+^, Cu^2+^, and Cd^2+^, while polypyrrole/aluminum fumarate MOF composites demonstrated nearly complete Pb^2+^ removal across a broad pH range.^174,225,227^ These findings underscore MOF-based nanocomposites as promising next-generation adsorbents for water treatment.

Despite these advances, several challenges hinder the transition of MOFs from laboratory demonstrations to large-scale applications. Economic feasibility is a primary concern, as MOF synthesis often requires costly high-purity linkers and metal precursors, along with energy-intensive solvothermal processes, making them more expensive than traditional adsorbents. To overcome this, researchers are exploring greener and scalable synthesis methods such as mechanochemical, microwave-assisted, and solvent-free routes, which can lower and reduce environmental impact.^225^ Structural stability in real wastewater systems presents another barrier. While some frameworks exhibit good hydrothermal stability, many MOFs degrade under strongly acidic or alkaline conditions, potentially leading to framework collapse and release of toxic metal nodes (*e.g.*, Cd, Cr, Co, Ag) or harmful organic linkers (*e.g.* 4,4′-bipyridine, pyrazine) during. The presence of competing ions and natural organic matter further reduces efficiency and lifespan. Addressing these issues requires the use of robust frameworks with strong metal–ligand bonds (*e.g.*, Zr-based MOFs) and the development of hybrid composites that improve structural integrity.^174,225^ Process engineering considerations also limit practical applications. Fine MOF powders, though effective in batch adsorption, are unsuitable for continuous-flow systems due to high pressure drops and potential material loss. Thus, MOFs must be formulated into pellets, granules, or membranes, but these processes often reduce accessible active sites. In addition, regeneration efficiency varies widely among frameworks, and large-scale production still requires substantial solvent and energy input, raising sustainability concerns.^225–229^ Nevertheless, research progress is steadily bridging the gap between laboratory innovation and industrial application. The development of more stable and cost-effective MOFs, combined with greener synthesis methods and improved formulation strategies, suggests a promising future for these materials in sustainable water treatment. Furthermore, integration with complementary technologies could further enhance their applicability.^174,212,225,227^

### 7.7 Layered double hydroxide-based adsorbents

Layered double hydroxides (LDHs) are two-dimensional anionic clays composed of positively charged brucite-like layers of divalent and trivalent metal cations, intercalated with water molecules and exchangeable anions. Their lamellar architecture provides adjustable interlayer spacing, high surface area, and significant anion exchange capacity, making them especially effective for adsorption of heavy metal cations. LDHs can be synthesized by cost-effective methods including co-precipitation, hydrothermal treatment, ion exchange, urea hydrolysis, or structure reconstruction, offering flexibility in composition and crystallinity.^230–232^ Pristine LDHs already display respectable adsorption capacities, but surface modification, composite formation, intercalation with functional ligands, defect engineering, and hybridization with materials such as biochar, graphene oxide, carbon nanomaterials, Fe_3_O_4_, or polymers significantly enhance their efficacy. For example, an LDH/biochar composite using orange peel biochar exhibited a Cu(ii) adsorption capacity of ∼68 mg g^−1^, outperforming CNT-modified LDHs (∼44 mg g^−1^) and unmodified ZnFe-LDHs (∼25 mg g^−1^), primarily due to greater surface area and abundance of binding sites. In another study, CaAl-LDHs achieved particularly high capacities (∼592 mg g^−1^ for Cd(ii)) under optimized conditions, highlighting the potential of functionalized LDHs for heavy metal removal in highly contaminated waters. The adsorption mechanism in LDHs involves ion exchange (replacement of interlayer anions by heavy metal ions), electrostatic attraction, surface complexation and, in some cases, chelation with functional ligands. Defects in the LDH layers, such as oxygen vacancies, and substitution of metal cations (*e.g.*, replacing M^2+^ or M^3+^ with metals of different ionic sizes) can influence the chemical reactivity and significantly enhance ion exchange and binding efficiency. Intercalation of anions and functional molecules into interlayer gallery, surface functionalization, and defect engineering are effective strategies to introduce binding affinity, expand adsorption sites, and improve adsorption kinetics.^174,230^

Despite these advances, LDH-based adsorbents face important limitations when considering practical, large-scale applications. Key challenges include structural instability under extreme pH conditions (strongly acidic or alkaline), loss of crystallinity, aggregation of particles, and decreased performance in the presence of competing ions or natural organic matter. Additionally, functional ligands or intercalated anions can leach out or degrade over repeated cycles, reducing long-term stability. Synthesis methods involving surfactants or sacrificial templates may introduce toxicity or require multi-step, energy-intensive processes. The trade-off between material performance (capacity, rate) and synthesis complexity or cost remains a critical issue.^230,233^ To improve applicability, recent studies suggest multiple strategies: designing defect-rich LDHs with robust metal–cation frameworks, using benign intercalated anions or environmentally safe ligands, hybridizing LDHs with stable supports (*e.g.*, biochar, polymer matrices) to prevent aggregation and facilitate separation, and optimizing regeneration protocols (*e.g.*, mild acids, bases, or chelating agents) to preserve structural integrity. Some hybrid LDH composites have shown excellent regeneration capability, maintaining a high proportion of their adsorption capacity after several cycles.^174,231,232^ Overall, LDHs remain one of the most promising adsorbent families for heavy metal removal due to their tunable structure, abundant binding sites, and versatility. Nonetheless, future research must rigorously address their stability regeneration efficiency, scale-up synthesis, and performance under real wastewater conditions to bridge the gap between lab-scale demonstrations and sustainable industrial applications.

A comparative summary of adsorption capacities, optimum operating conditions, regeneration cycles, and kinetic parameters of representative nanoadsorbents is presented in Table 8, highlighting the relative performance of different material categories.

**Table 8:** Comparative performance of representative nanoadsorbents for heavy metal removal

Adsorbent	Heavy metal	Adsorption capacity (mg g^−1^)	pH	*T* (K)	Regeneration cycles	Kinetic parameters	Ref.
Ag–Fe MOF	Cd(ii)	265 – Langmuir	7	298	5	PSO − *K*_2_ = 0.000870	234
	Cu(ii)	213 – Langmuir	5			PSO − *K*_2_ = 0.002220	
CoMnMOF-74	As	531 – Langmuir	11	298	4	PSO − *K*_2_ = 0.01058	235
Polyaspartic acid-LDH	Pb(ii)	229.2 – Langmuir	6	303	4	PSO − *K*_2_ = 0.000166	232
	Hg(ii)	208.6 – Langmuir	4			PSO − *K*_2_ = 0.000133	
Fe_3_O_4_/cyclodextrin polymer	Ni(ii)	13.2 – Langmuir	5.5	298	4	PSO − *K*_2_ = 0.033	236
	Cd(ii)	27.70 – Langmuir				PSO − *K*_2_ = 0.016	
	Pb(ii)	64.50 – Langmuir				PSO − *K*_2_ = 0.003	
Polypyrrole–polyethyleneimine	Pb(ii)	75.60 – Langmuir	10	323	5	PSO − *K*_2_ = 0.031	237
Surfactant/nano zeolite	Pb(ii)	91.34 – Freundlich	6	318	NR	NR	238
	Cu(ii)	85.71 – Freundlich				NR	
	Cd(ii)	78.27 – Langmuir				NR	
	Ni(ii)	76.18 – Freundlich				NR	
	Zn(ii)	67.41 – Freundlich				NR	
	Fe(ii)	63.45 – Freundlich				NR	
Magnetic nano zeolite	Cu(ii)	59.9 – Langmuir	7	298	NR	PSO − *K*_2_ = 0.000179	239
	Cd(ii)	188.6 – Langmuir				PSO − *K*_2_ = 0.000072	
	Pb(ii)	476.1 – Langmuir				PSO − *K*_2_ = 0.000024	
Graphene oxide–starch	Cu(ii)	542.01 – Freundlich	8	283	3	PSO − *K*_2_ = 0.0.0206	240
Activated carbon	Ni(ii)	19.19 – Langmuir	12	313	NR	PSO − *K*_2_ = 0.0.00265	241
	Cr(vi)	47.42 – Langmuir	2	310	NR	PSO − *K*_2_ = 0.0.000152	
nZVI	Cu(ii)	78.38 – Freundlich	5	303	6	PSO − *K*_2_ = 0.0.00000103	242
Silver nanoparticles and magnetic nanoparticles/nanocomposites	Pb(ii)	23.75 – Langmuir	5	298	3	PSO − *K*_2_ = 0.0387	243
	Ni(ii)	12.38 – Langmuir	6			PSO − *K*_2_ = 0.0051	
	Cu(ii)	18.57 – Langmuir				PSO − *K*_2_ = 0.0094	
	Cd(ii)	8.71 – Langmuir				PSO − *K*_2_ = 0.0039	
Nano SiO_2_	Pb(ii)	117.74 – Freundlich	5	298	6	PSO − *K*_2_ = 0.015	224
	Cr(vi)	51.42 – Freundlich	2			PSO − *K*_2_ = 0.008	

## 8. Recycling and regeneration of nanoadsorbents

The economic feasibility and environmental sustainability of nanoadsorbent-based water treatment technologies are fundamentally determined by their ability to be regenerated and reused effectively. Reusability is defined as the capacity of an adsorbent to retain its structural integrity and adsorption efficiency after several adsorption–desorption cycles, thus minimizing material waste and operational costs while enhancing environmental compatibility.^62,244–249^ Despite the promising adsorption capabilities and surface functionalities exhibited by various nanoadsorbents, their large-scale application remains hindered by challenges associated with recyclability, structural deterioration, and declining performance upon repeated use. One of the foremost operational challenges in nanoadsorbent recycling is the efficient separation and recovery of nanosized particles from treated effluents. The extremely small particle size and high dispersibility of nanomaterials complicate conventional recovery methods such as filtration or sedimentation, which often fail to achieve quantitative recovery.^62,244^ Incorporating membrane-assisted or hybrid separation systems can improve recovery but adds substantial complexity and cost. Moreover, incomplete recovery may lead to the unintended release of residual nanomaterials into water bodies, raising significant ecotoxicological and health concerns.^244,248^ Magnetic nanoadsorbents have emerged as a promising strategy to overcome these limitations, enabling rapid and energy-efficient recovery under an external magnetic field.^245,249^ For example, Fe_3_O_4_-based nanocomposites and Fe@Au–graphene oxide hybrids have shown efficient magnetic separation and stable adsorption performance over multiple cycles, highlighting the potential of magnetically assisted recycling.^245^ However, their long-term stability under varying pH, salinity, and redox conditions remains a key concern, as environmental fluctuations can alter magnetic and surface properties, leading to performance degradation.^246,249^

Another critical issue in the regeneration of nanoadsorbents is the progressive decline in adsorption capacity with successive reuse cycles. This reduction is primarily attributed to irreversible surface fouling, pore blockage by organic or inorganic matter, and loss of active sites or functional groups during regeneration.^246,249^ For nanofibrous and porous materials, repeated exposure to wastewater can also induce shrinkage, deformation, and microstructural weakening, compromising both mechanical stability and adsorption efficiency.^244^ The extent of performance deterioration is strongly influenced by the regeneration technique employed, necessitating optimization of the regeneration method to balance desorption efficiency, structural preservation, and environmental safety.

Chemical regeneration using acids, bases, or chelating agents remains the most common approach due to its simplicity and high adsorption efficiency. However, the choice and concentration of regenerants critically determine the degree of adsorbent preservation. For instance, Fe/Cu nanoparticles exhibited higher As(iii) desorption efficiency under alkaline conditions, while acidic eluents were more effective for As(v). Similarly, EDTA-functionalized Fe_3_O_4_ nanoparticles achieved up to 98% regeneration efficiency using 0.5 M HCl, maintaining performance across 30 reuse cycles.^245^ Despite such successes, aggressive chemical treatments can lead to dissolution of active components, leaching of metals, or destruction of surface functionalities. Thermal and electrochemical regeneration methods provide alternative routes, though each has inherent drawbacks; thermal treatments can induce sintering and surface area loss, while electrochemical methods require high energy input and specialized electrodes.^248^ Regardless of the regeneration method, managing the fate of desorbed pollutants remains a major environmental concern, as inadequate handling can lead to secondary contamination.^248,250^ Therefore, the development of closed-loop regeneration systems capable of capturing and neutralizing desorbed contaminants is essential for safe and sustainable implementation.

**Fig. 22:**
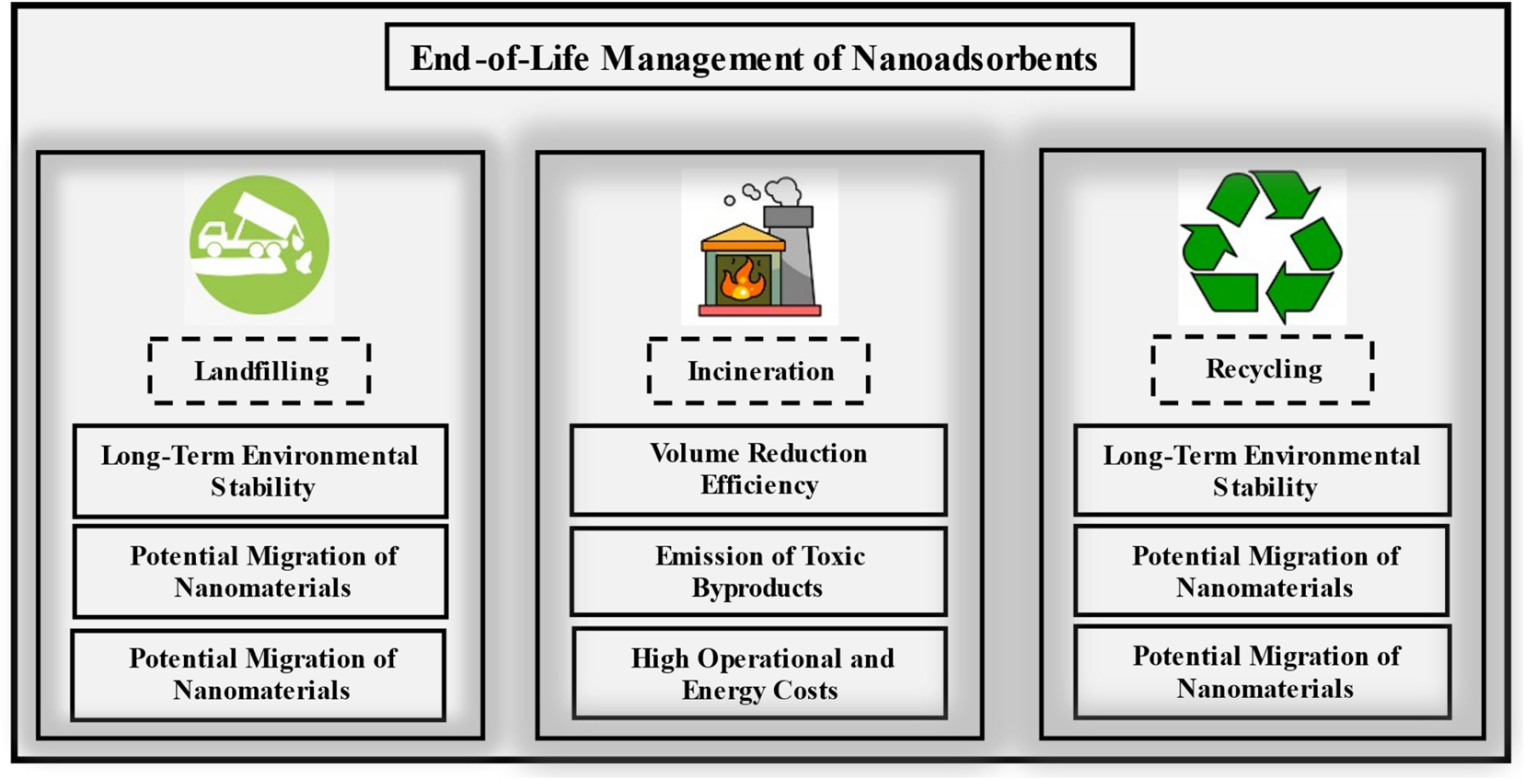
End-of-life management of nanomaterials, highlighting environmental impacts and challenges associated with landfilling, incineration, and recycling.^248^

Beyond the regeneration process itself, the environmental and lifecycle implications of nanoadsorbent recycling must be critically considered. The high reactivity and small size that make nanomaterials effective adsorbents also enhance their mobility and persistence in ecosystems if released inadvertently. Leaching of nanoparticles or transformation under variable environmental conditions can lead to bioaccumulation, toxicity, and disruption of microbial communities. To mitigate such risks, encapsulation within inert matrices, surface functionalization to reduce reactivity, and integration into composite systems have been proposed. End-of-life management further presents challenges: landfilling risks nanoparticle leaching; incineration may release toxic emissions; and recycling requires energy-intensive processes that must be optimized to remain economically viable.^248,250^Fig. 22 illustrates the key environmental and operational challenges associated with various end-of-life management routes for nanomaterials, including landfilling, incineration, and recycling. Therefore, recycling technologies should be designed within the framework of circular economy principles, emphasizing material recovery, safe disposal, and minimal environmental footprint.

From a regulatory and practical perspective, the standardization of nanoadsorbent recycling practices remains insufficient, with inconsistent safety and performance guidelines across jurisdictions.^249,251^ Establishing harmonized international standards for recyclability, regeneration protocols, and environmental compliance will be critical to support broader adsorption and commercialization. Future research should prioritize green regeneration technologies, such as low-energy electrochemical systems, solvent-free desorption, and biodegradable nanocomposites, which minimize both cost and environmental burden.^246,247,249^ Moreover, the exploration of alternative reuse pathways for spent nanoadsorbents, such as their utilization in catalysis, soil conditioning, or as additives in cementitious materials, offers sustainable solutions that extend their functional lifespan while reducing waste generation.^250^ Overall, while the regeneration and reuse of nanoadsorbents hold significant promise for enhancing the sustainability and cost-effectiveness of water purification technologies, numerous technical, environmental, and regulatory challenges must be addressed before large-scale implementation. Achieving this goal requires interdisciplinary collaboration that bridges materials science, environmental engineering, toxicology, and policy development to ensure that next-generation nanoadsorbents are both highly efficient and environmentally responsible throughout their entire life cycle.^244–249^

## 9. Conclusions

Water pollution, especially from heavy metals, continues to be a significant worldwide concern owing to the persistence, non-biodegradability, and bioaccumulative characteristics of these contaminants. Rapid industrialization, urban growth, and human activities have considerably affected the contamination of water resources, promoting the need for effective, sustainable, end economical treatment approaches. Conventional water treatment techniques, while widely utilized, often inadequately address the complex chemical behavior and low concentrations of heavy metals in aquatic environments. This has promoted considerable interest in adsorption approaches using nanomaterials because of their exceptional surface characteristics, adjustable functionality, and improved reactivity.

Nanotechnology offers an innovative method for environmental remediation. Engineered nanomaterials, including carbon-based nanoadsorbents, zeolites, metal and metal oxide nanoparticles, polymer-based nanocomposites, metal–organic frameworks (MOFs), and layered double hydroxides (LDHs), exhibit exceptional adsorption capacities and selectivity for a variety of heavy metal ions. Their enhanced surface area-to-volume ratio, adjustable structure, and capacity for functionalization make them very efficient in targeting particular pollutants, even at trace concentrations.

**Fig. 23:**
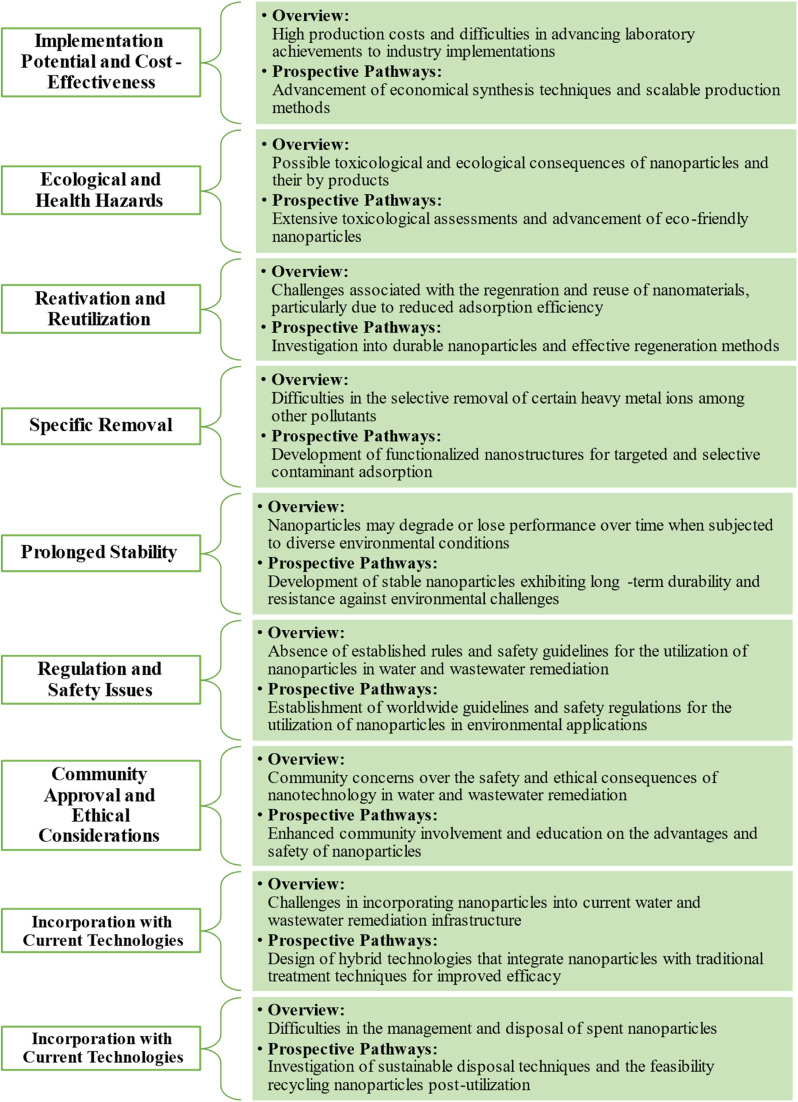
Main issues and prospective pathways for the utilization of nanoparticles in the removal of heavy metal ions from water and wastewater.^212^

Nonetheless, despite these benefits, several obstacles prevent the widespread utilization of nanoadsorbents. This involves the scalability of synthesis, economic viability, long-term stability, regeneration and reusability, toxicity of nanomaterials, and environmental fate issues. Fig. 23 demonstrates the principal obstacles currently limiting the extensive use of nanomaterials in water and wastewater treatment and provides prospective research pathways to address these issues. This encompasses the development of economical and environmentally friendly synthesis processes, enhancement of regeneration methods, optimization of adsorption selectivity across diverse environmental conditions, and execution of thorough toxicity evaluations.

Furthermore, extensive pilot and industrial studies are critically required to validate laboratory findings, assess economic feasibility, end evaluate environmental effects in real-world situations. The advancement of real-time performance assessment tools and intelligent monitoring systems combined with Internet of Things (IoT) technology might significantly improve the operation efficiency and traceability of nanomaterial-based water treatment systems.^212^

Despite their remarkable potential for environmental remediation, several challenges hinder the large-scale application of nanomaterials. Nanoparticle leakage poses significant environmental risks, as released particles may accumulate in ecosystems, interact with microorganisms, and induce oxidative stress or toxicity. Ensuring nanoparticle stability, recovery, and safe disposal is therefore essential. Another major concern is regeneration efficiency many nanomaterials lose adsorption capacity after repeated use due to surface fouling, oxidation, or structural degradation. Developing durable and easily regenerable materials, such as magnetically separable or composite nanostructures, can help mitigate this issue. Furthermore, comprehensive life cycle analysis (LCA) is required to evaluate the overall sustainability of nanomaterials, taking into account their synthesis, operational performance, and disposal impacts. Finally, high production and regeneration costs remain a major barrier to commercialization, emphasizing the need for scalable, low-cost, and green synthesis strategies. Addressing these challenges is crucial to achieving safe, efficient, and economically viable nanomaterial-based water treatment technologies.

## Author contributions

E. B. Hussein: conceptualization; methodology; data curation; formal analysis; investigation; resources; writing – original draft; writing – review & editing; visualization; project administration. A. S. Mohammed: formal analysis; investigation; writing – review & editing; visualization. F. A. Rasheed: formal analysis; investigation; writing – review & editing; visualization. K. F. Kayani: formal analysis; investigation; writing – review & editing; visualization.

## Conflicts of interest

There are no conflicts to declare.

## Data Availability

No primary research results, software or code have been included and no new data were generated or analysed as part of this review. Data sharing is not applicable to this article as it is a review of previously published literature, and all supporting data are available in the cited references.
